# Directed evolution of a cytochrome P450 monooxygenase for improved perillyl alcohol biosynthesis *via* a tailored genetically encoded biosensor

**DOI:** 10.1039/d6ra03129c

**Published:** 2026-07-03

**Authors:** Catherine A. Odhiambo, Alexandra A. Malico, Gavin J. Williams

**Affiliations:** a Department of Chemistry, NC State University Raleigh North Carolina 27695 USA gjwillia@ncsu.edu; b Comparative Medicine Institute, NC State University Raleigh North Carolina 27695 USA

## Abstract

Perillyl alcohol is a naturally occurring terpene with promising anticancer properties. However, like other oxidized terpenes, its limited biosynthetic availability hinders its development as a therapeutic lead. Cytochrome P450 monooxygenase CYP153A6 catalyzes the key hydroxylation of limonene to yield perillyl alcohol in *Escherichia coli*, yet the enzyme is the rate-limiting step in this pathway and has not previously been improved by directed evolution. Here, we report the development of a genetically encoded biosensor based on the cymene repressor CymR from *Pseudomonas putida* F1, engineered to selectively detect perillyl alcohol over its non-hydroxylated precursor limonene, and its application to the directed evolution of CYP153A6. A single substitution (S77L) in the CymR effector-binding pocket dramatically increased biosensor sensitivity and dynamic range toward perillyl alcohol while maintaining discrimination against limonene. The tailored CymR-S77L biosensor was coupled to a CYP153A6 random mutant library to enable high-throughput fluorescence-based screening of variants. Ultimately, seven CYP153A6 variants, some with multiple mutations, were identified to have improved perillyl alcohol titers. Deconvolution of beneficial mutations revealed that a single substitution, A287T, located near the predicted CYP153A6:ferredoxin reductase interface, was sufficient to increase perillyl alcohol productivity 4.2-fold relative to wild-type without compromising regio- or chemoselectivity. These results demonstrate that biosensor-guided directed evolution is an effective and selective strategy for engineering P450-dependent terpene hydroxylation pathways and establish a generalizable platform for developing monoterpene-responsive biosensors and applying them to the directed evolution of terpene-modifying enzymes.

## Abstract 2

A tailored CymR-S77L biosensor enabled high-throughput evolution of a P450 for selective perillyl alcohol production from limonene, establishing a platform for terpene hydroxylation engineering.

## Graphical abstract

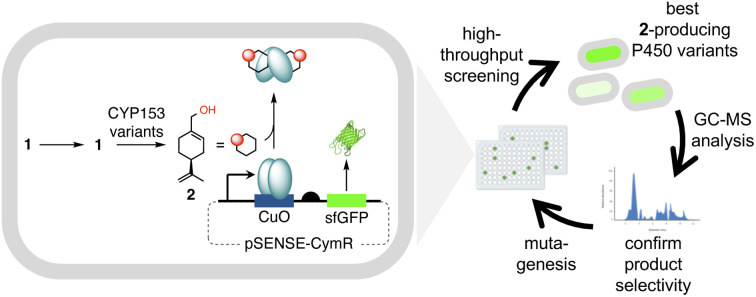

## Introduction

Cytochrome P450 monooxygenases (P450s) are among the most versatile and industrially significant enzyme families in nature, for which approaches to engineer them are highly sought after. P450s facilitate a broad range of oxidative reactions, including hydroxylation, epoxidation, dealkylation, and heteroatom oxidation.^1,2^ Their broad substrate scope and ability to introduce oxygen into unactivated C–H bonds make them especially valuable for synthetic biology, drug metabolism, and natural product biosynthesis, where they attract significant interest as a complement to traditional organic synthesis.^3,4^ In terpene biosynthesis, P450s are crucial for creating structural diversity by controlling regio- and stereoselective hydroxylation, which directly influences biological activity and downstream derivatization potential.^5,6^ However, P450s often show low activity, inefficient electron use, and limited compatibility with heterologous hosts such as *E. coli*.^3,7–9^ Many P450s are not optimized for non-native substrates or reactions, are often rate-limiting in their native pathways, and many that catalyze suspected reactions remain unidentified. These limitations pose a significant barrier to the use of P450s as scalable biocatalysts for terpene functionalization.

**Fig. 1:**
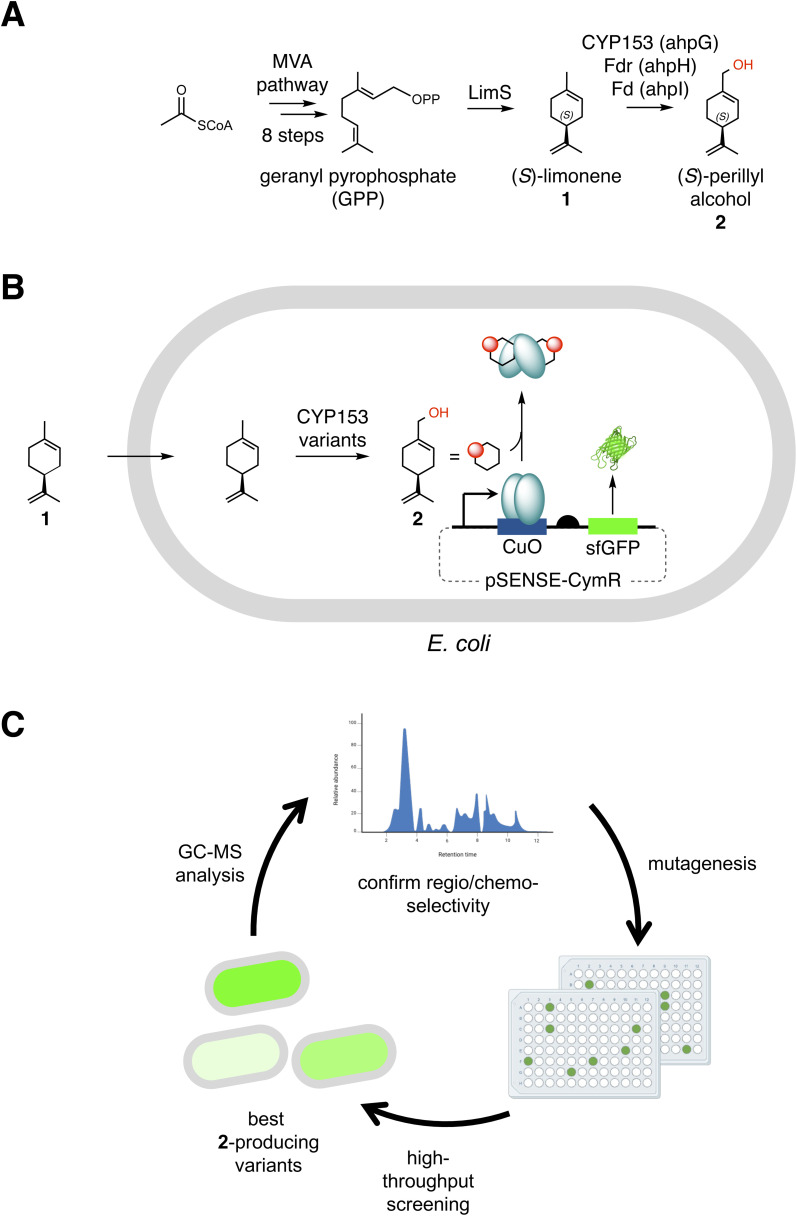
P450-mediated biosynthesis of 2. (A) P450-mediated oxidation biosynthetic route to 2*via* GPP and the terpene synthase, LimS. (B) Reporting the P450-mediated conversion of 1 to 2*via* a transcription-factor-based biosensor. (C) A 2-selective transcription factor biosensor enables directed evolution of CYP153A6.

For example, perillyl alcohol (*e.g.* (*S*)-perillyl alcohol, 2, Fig. 1a), is a natural monoterpene that has attracted sustained interest due to its promising anticancer activity, ability to cross the blood–brain barrier with minimal systemic toxicity, versatile scaffold for synthesis of derivatives with enhanced bioactivities, and potential other bioactivities, for which engineered P450s could facilitate access.^10–24^ However, the limited availability of 2 has hindered its development as an anticancer lead.^23^ Chemical synthesis of 2*via* hydroxylation of 1 requires four to six steps and typically yields product mixtures.^23,25^2 has been biosynthesized through ten enzymatic steps (Fig. 1a) involving geranyl pyrophosphate synthase (GPPS), limonene synthase (LimS), and a site-specific hydroxylation reaction catalyzed by a cytochrome P450 monooxygenase.^26–32^ One well-characterized P450 system for producing 2 utilizes the membrane-bound alkane hydroxylase P450 CYP153A6 (AhpG), its associated reductase (RED, AhpH), and ferredoxin (Fd, AhpGI) from *Mycobacterium* sp. HXN-1500 (Fig. 1a).^27,33,34^ However, the P450 is likely the rate-limiting step in 2 biosynthesis in *E. coli*,^32^ and its activity with 1 has yet to be improved by enzyme engineering.

The directed evolution of CYP153A6 could address the need for an improved enzyme for 2-production, especially given the lack of structural and mechanistic information for this enzyme. Efforts to engineer P450s through rational design and directed evolution^9,35,36^ rely on traditional screening methods, such as GC-MS, that are inherently low-throughput and labor-intensive.^37–39^ Other approaches are limited in scope and throughput.^40^ For terpene oxidation in particular, the challenge is amplified by the potential for competing regio- and stereochemical outcomes and over-oxidation. The directed evolution of P450s for selective terpene functionalization remains comparatively limited, underscoring the need for additional screening strategies, particularly high-throughput approaches.^9^

Genetically encoded biosensors are powerful tools for enzyme-directed evolution by enabling rapid, high-throughput detection of *in vivo* metabolites *via* diverse readouts.^41–43^ Although many transcription factor–analyte pairs have been developed,^44–50^ only a few for select monoterpenes have been described.^51–53^ Furthermore, none have been developed for terpene-modifying P450s.

Herein, we set out to use the cymene repressor (CymR) from *Pseudomonas putida* F1 ^54^ as a platform to develop a genetically encoded biosensor for reporting the P450-catalyzed biosynthesis of 2 (Fig. 1b). The biosensor was developed to selectively detect the hydroxylated product (2) while discriminating against its non-oxidized precursor (1). This enabled the identification of P450 variants (Fig. 1c) with significantly higher productivity for the target product, confirming P450-focused biosensor-guided evolution as an effective platform for engineering terpene hydroxylation pathways.

## Experimental

### Bacterial strains, plasmids, and materials

Synthetic oligonucleotides (SI Table S1) were purchased from Integrated DNA Technologies (Coralville, IA, USA). Enzymes used for DNA manipulation and plasmid isolation *via* a mini-prep kit were purchased from New England Biolabs (Ipswich, MA, USA). Polymerase chain reactions were carried out with Q5 Hot Start High Fidelity 2X master mix. Plasmid sequences were verified by DNA sequencing of the target gene (Azenta, Research Triangle Park, NC, USA) or the full-length plasmid (Plasmidsaurus, Louisville, KY, USA).

The *E. coli* DH5α and TOP10 strains (Invitrogen) were used for standard cloning procedures and maintenance of genetic constructs. Recombinant protein expression was performed in BL21(DE3) cells. Unless otherwise stated, all solids were dissolved in 18.2 mΩ resistance H_2_O ELGA water. A 10x dry PBS from APEX® was used to prepare a final (1x) solution for resuspending the cell pellet. Commercial *S*-(−)-limonene (1), used as a ligand in the bioconversion assays, was purchased from Sigma-Aldrich and prepared in dimethyl sulfoxide (DMSO) to a stock concentration of 10–250 mM. Absorbance and fluorescence readings were taken in clear flat-bottom and black flat-bottom 96-well plates (Greiner Bio-One®), respectively. The readings were taken using a BioTek Hybrid Synergy 4® plate reader. Ethyl acetate was used to extract the target biosynthesis product *S*-(−)-perillyl alcohol (2). Reagents used were reagent grade or better.

### Homology modeling of CymR and docking studies

The default MM2 energy-minimization parameters in ChemDraw 3D were used to optimize terpene 2 for docking. It was saved in mol2 format and then converted to pdbqt using OpenBabel.^55^ The CymR homology model was prepared for docking in AutoDockTools.^56–58^ Docking constraints were set for the putative ligand-binding site based on other TetR-type repressor proteins. AutoDock Vina was used to dock the ligand and receptor. Results were generated with a grid box of 64 × 50 × 42, grid spacing of 1.00, and an exhaustiveness of 25, centered at coordinates 32 × −8 × 50 to ensure the entire CymR model receptor was encompassed within the grid box, and the results were analyzed in PyMOL.^58^

### Dose–response analysis of wild-type and variant CymR biosensor strains

Similar to our previous analysis of CymR variants,^52^ freshly transformed single colonies of the wild-type or variant CymR biosensor strains in *E. coli* BL21(DE3) cells were picked from LB agar plates and used to inoculate 20 mL of LB containing 100 µg mL^−1^ ampicillin. Cultures were incubated at 37 °C with shaking at 350 rpm for 5 h. A 495 µL volume of culture was transferred to a deep 96-well plate and induced with equal volumes (5 µL) of the terpenes (1 and 2) in the appropriate wells, yielding final concentrations ranging from 0.1 to 2.5 mM. For controls, 5 µL of DMSO was added to three wells corresponding to each CymR variant. The plates were covered with ThermalSeal wrap and incubated for 16 h at 37 °C with shaking at 350 rpm. The cells were harvested by centrifugation at 1509×*g* and 4 °C for 10 min, then resuspended in 600 µL of 1X phosphate-buffered saline (PBS), pH 7.5. The cell suspension (100 µL) was transferred to clear and black flat-bottom 96-well plates to analyze the OD_600_ and fluorescence (ex 485 nm/em 510 nm), respectively. Normalized GFP fluorescence values were obtained by dividing fluorescence intensity by the corresponding OD_600_ values. This is followed by the subtraction of the normalized fluorescence output at zero ligand (relative fluorescence). Concentrations and relative fluorescence intensities were plotted and analyzed *via* the Hill equation with GraphPad Prism 10.
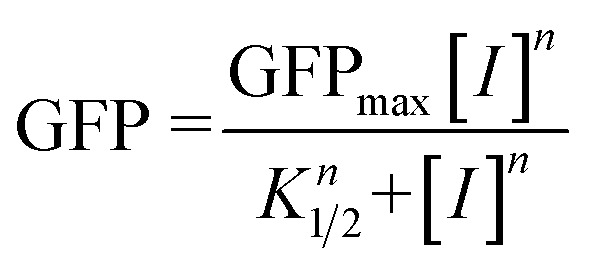
Here, GFP_max_ is the maximum normalized GFP expression, [*I*] is the effector concentration, *n* is the Hill coefficient that quantifies the cooperativity of the protein, and *K*_1/2_ is the effector concentration at half-maximal normalized fluorescence.

### General procedure for microplate screening of CymR variants

Similar to our previous CymR work,^52^ single colonies from mutant libraries in *E. coli* BL21(DE3), along with wild-type or parent strains, were picked from LB agar plates supplemented with 100 µg mL^−1^ ampicillin and used to inoculate 500 µL of LB media containing 100 µg mL^−1^ ampicillin in deep 96-well microplates. The cultures were covered by Aeraseal and incubated for 16 h at 37 °C with shaking at 350 rpm. Aliquots (5 µL) of the 16 h culture were transferred to each corresponding well, which contained 490 µL of LB media with 100 µg mL^−1^ ampicillin, covered with Aeraseal, and then incubated for 5 h at 37 °C with shaking at 350 rpm. One microplate was induced by adding 5 µL of 1 to a final concentration of 0.5 mM, and the other received 5 µL of DMSO. The two microplates were covered with ThermalSeal and incubated for an additional 16 h at 37 °C with shaking at 350 rpm. The cultures were centrifuged at 1509×*g* for 10 min, after which the cell pellet was resuspended in 600 µL of 1X phosphate-buffered saline (PBS). Two 100 µL aliquots of each cell suspension were transferred into a transparent Greiner 96-well microplate and a black Greiner 96-well microplate. The optical density of the cells was analyzed at 600 nm using the transparent microplate, and the fluorescence was measured at 485 nm excitation and 510 nm emission using the black microplate. The fluorescence was divided by the OD_600_ to yield a normalized GFP fluorescence value.

### CymR-guided high-throughput screening of the CYP153A6 mutant library

Single colonies of the CYP153A6 error-prone PCR library members in the dual plasmid strain containing the mutant biosensor plasmid (CymR-S77L) and the 2-production platform (P450 library and Fdr/Fd) in *E. coli* BL21(DE3) were picked from LB agar plates into wells of a 96-well microplate containing LB media supplemented with ampicillin and kanamycin each at 50 µg mL^−1^. The deepwell microplates were covered with Aeraseal and incubated for 16 h at 37 °C with shaking at 350 rpm. Aliquots of 5 µL from the overnight cultures were transferred to two separate 96-well microplates, each containing 490 µL of LB media supplemented with ampicillin and kanamycin, each at 50 µg mL^−1^, covered with Aeraseal, and incubated for 5 h at 37 °C with shaking at 350 rpm. The samples were then induced by adding 5 µL of isopropyl-β-d-thiogalactopyranoside (IPTG) to reach a final concentration of 0.5 mM. To one of the two 5 h deep well microplates, 5 µL of 1 was added to a final concentration of 0.5 mM, while 5 µL of DMSO was added to the other microplate. The two microplates were sealed with ThermalSeal wrap and incubated for 16 h at 37 °C with shaking at 350 rpm. Cells were harvested *via* centrifugation at 1509×*g* at 4 °C for 10 min, then resuspended in 600 µL of phosphate-buffered saline (1X PBS), pH 7.5. A 100 µL aliquot of the cell suspension was transferred to clear and black flat-bottom 96-well microplates for OD_600_ and fluorescence analysis (ex 485 nm/em 510 nm), respectively. Normalized GFP fluorescence values were calculated by dividing fluorescence intensity by the corresponding OD_600_ value.

For secondary screening, highly fluorescent colonies from the primary screen were picked and grown for 5 h with the appropriate antibiotic. After which, they were induced by adding IPTG to a final concentration of 0.5 mM. To one of the two 5 h growth cultures, 5 µL of 1 was added to a final concentration of 0.5 mM; the culture was sealed with a thermal seal wrap and incubated for 16 h at 37 °C with shaking at 350 rpm. The activity of the clones was analyzed *via* GC-MS. For tertiary screening, clones with higher productivity were isolated from the biosensor *via* kanamycin (50 µL) selection, and whole-plasmid sequencing was performed. The isolated CYP153A6 variants were separately transformed into chemically competent *E. coli* BL21 Star (DE3). The transformation process involved a heat shock step at 42 °C for 50 s, followed by a recovery phase in 950 µL of SOC medium at 37 °C for 1 h while shaking at 250 rpm. After recovery, a 25 µL aliquot of the transformation mixture was plated on LB agar containing 50 µg mL^−1^ kanamycin and incubated for 16 h at 37 °C. Two constructs were used as controls: one was wildtype, representing the CYP153A6 system, and the second was a construct lacking the P450 enzyme (pACYCDuet-RED-Fd).

### GC-MS analysis of wild-type and variant CYP153A6 strains

Similar to our previous work,^52^ a 300 µL aliquot of culture was transferred into separate Eppendorf tubes, and an equal volume of ethyl acetate was added. This was followed by centrifugation at 4816×*g* for 10 min at 25 °C. The organic layer was removed, dried over MgSO_4_, and transferred to glass tubes for GC-MS analysis. GC-MS analysis was conducted on an Agilent 8860 using a J&W CycloSil-B, with a 30 m × 250 µm × 0.25 µm capillary column. Helium (99.99% purity) was used as a carrier gas at a flow rate of 1.2 mL min^−1^. The oven was programmed to start at 50 °C for 3 min, then increase at 25 °C min^−1^ to 100 °C, followed by a 10 °C min^−1^ increase to 140 °C, and finally a 20 °C min^−1^ increase to 250 °C. The injection port was set at 250 °C on pulsed splitless mode. The injection volume was 1 µL. The EI mode 70 eV. The mass data were acquired after a 4.2 min solvent delay in scan mode, ranging from 30 to 500 *m*/*z*.

### Modeling of the CYP153A6 protein structure

The CYP153A6 sequence was submitted to the AlphaFold^59^ and I-TASSER^60^ servers, and a homology model was constructed. The catalytic sites of these enzymes were predicted based on the available crystal structure (PDB: 3RWL),^61^ and the results were analyzed in PyMOL.^58^

### Computational prediction of the CYP153A6/Fdr/Fd complex

Substrate binding channels were identified using MOLEonline.^62^ The CYP153A6 ternary complex was predicted using the AlphaFold 2/AlphaFold Multimer modules^59,63^ within ChimeraX.^64^

## Results and discussion

### Development of a prototype CymR-based biosensor for reporting the hydroxylation of 1

**Fig. 2:**
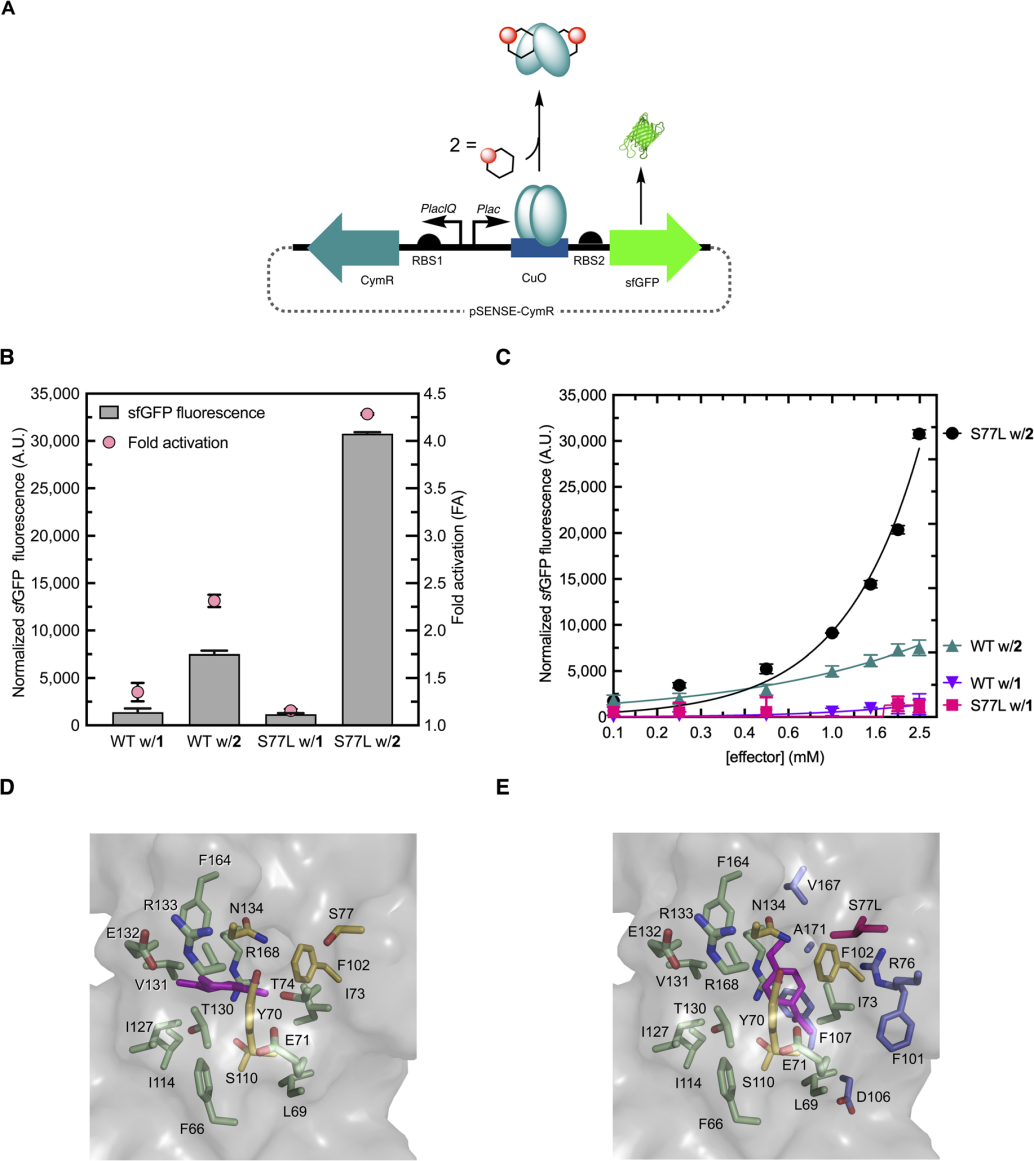
Performance of wild-type and CymR-S77L with 1 and 2. (A) Scheme illustrating the CymR synthetic genetic circuit. (B) Normalized GFP fluorescence of the WT and S77L biosensor strains with 2.5 mM of 1 and 2 and fold activation. (C) Dose–response curves of WT and S77L with varying concentrations of 1 and 2. Error bars (where visible) represent the standard error of the mean (*n* = 3). (D) Wild-type CymR homology model with 2 (magenta sticks) docked. Residues within a 5 Å radius of the ligand are shown as green sticks, with those targeted for saturation mutagenesis shown in yellow (Tyr70, Ser77, Phe102, Ser110, and Asn134). (E) Model of CymR S77L with 2 docked. Residues within a 5 Å radius of the ligand are shown as blue sticks, with those targeted for saturation mutagenesis shown in yellow (Tyr70, Phe102, Ser110, and Asn134), and S77L is shown in magenta. Residues within a 5 Å radius of the ligand in the wild-type model are shown as green sticks.

To initiate the development of a biosensor platform for detecting 2, a prototype genetic circuit incorporating wild-type CymR was constructed from a previously reported plasmid, yielding pSENSE2-CymR (Fig. 2a; SI Table S2 and Fig. S1).^49^ The repressor module consisted of a codon-optimized CymR from *Pseudomonas putida* F1, constitutively expressed under the *P*_lacI_^q^ promoter. The reporter module consisted of superfolder GFP (*sf*GFP), whose expression is controlled by a single cognate operator sequence (cuO) positioned downstream of the *sf*GFP promoter (*P*_*sf*GFP_). In the unbound condition, CymR interacts with CuO. Upon binding of the effector, CymR releases CuO, allowing transcription and translation of a reporter gene (Fig. 2a). Dose–response analysis of the wild-type CymR biosensor showed that it responded to compound 2 poorly (Fig. 2b and c), highlighting the need for biosensor engineering to enable selective detection.

To guide protein engineering of CymR toward recognition of 2, a docking model of 2 bound in the effector-binding pocket was examined (Fig. 2d). Amino acid residues within 5 Å of the docked ligand were identified. One residue from each of several structurally distinct regions of the pocket was selected as a candidate site for mutagenesis (Tyr70, Ser77, Phe102, Ser110, and Asn134). Five residues (Tyr70, Ser77, Phe102, Ser110, and Asn134) were selected as candidates for mutagenesis. Saturation mutagenesis libraries were created for these residues, and a concurrent microplate screen in *E. coli* using 1 and 2 identified S77L as the top-performing variant.

Interestingly, although the leucine introduced at position 77 is within a 5 Å radius of residues around the ligand, it is not the closest compared to other side chains (Fig. 2e). Instead of forming a primary contact, Leu77 sits at the edge of the effector binding environment, likely contributing to the pocket's overall hydrophobic character rather than directly affecting the ligand's placement. This suggests that the effect of the S77L substitution likely comes from more subtle changes, such as altering local packing or pocket flexibility, rather than creating new close–range interactions with the ligand itself.

To evaluate whether CymR can act as a dependable reporter for P450-mediated hydroxylation and specifically detect the desired product 2 over its biosynthetic precursor 1, we measured the fluorescence response of the pSENSE2-CymR circuit across a range of ligand concentrations (Fig. 2b and c). Cultures expressing wild-type CymR showed only a limited response to 2, with modest changes in reporter output and no clear dose–response between 0.1 and 2.5 mM. Introducing the S77L substitution greatly enhanced the response profile. This variant exhibited a pronounced induction curve with a substantially higher dynamic range (30 800 ± 160) and fold activation (4.3 ± 0.01) compared to wild type, which had a response of 7500 ± 340 and a fold activation of 2.3 ± 0.06 (Fig. 2b and c). Notably, the CymR-S77L variant remained unresponsive to 1 across the entire concentration range tested; specifically, its response was ∼26 times higher at 2.5 mM of 2 than to 1. This demonstrates that the engineered biosensor has increased sensitivity to 2 while still effectively discriminating against the precursor structure. This was further highlighted by the improved ability of CymR S77L to report the concentration of 2 in mixtures with 1, mimicking the CYP153A6 conversion in the biosensor strain (SI Fig. S2).

### Effector specificity of wild-type CymR and S77L

**Fig. 3:**
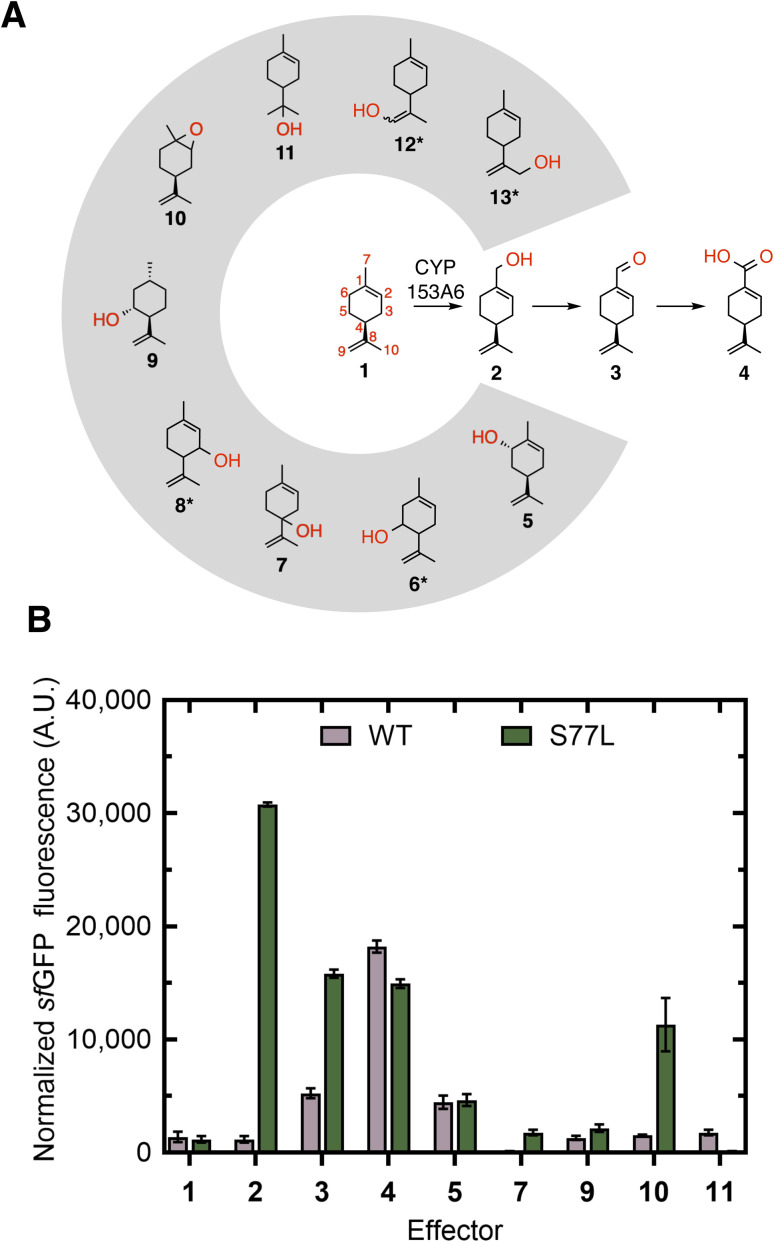
Effector profile of the wild-type and S77L CymR biosensor strains. (A) Possible products from P450-based oxidation of 1. Structures labeled with an asterisk were not available for testing. (B) Normalized GFP fluorescence (corrected by subtracting fluorescence with no effector) of wild-type and S77L with available monoterpenes at 2.5 mM. Error bars represent the standard error of the mean (*n* = 3).

To understand how the S77L substitution alters effector recognition, we tested both CymR-WT and CymR-S77L against a set of oxygenated derivatives of 1 (3–13) at a concentration of 2.5 mM (Fig. 3a). Since iterative P450 oxidation can produce over-oxidized compounds, and CYP153A6 mutants might oxidize positions on 1 not targeted, the compound panel was intentionally crafted to include both over-oxidized derivatives at the target limonene hydroxylation sites (3, 4) and alternative mono-hydroxylated regio/stereoisomers resulting from oxidation at other positions on the limonene core (5–13). CymR-S77L showed higher response with several panel members compared to the wild-type biosensor (*e.g.*, 2, 3, 7, 9, 10; Fig. 3b). The target effector 2 showed the highest fluorescence among the terpenes tested (Fig. 3b). Interestingly, effectors 3, 4, and 5 demonstrated similar overall output between the wild-type and S77L CymR variants (Fig. 3b). Additionally, CymR S77L could distinguish epoxide 10 mutant from 1, as judged by the fluorescence response, unlike the wild-type biosensor. Although the CymR biosensor does not totally distinguish between 2 and the over-oxidized compounds 3, 4, 10, the derivatives are easily differentiated based on mass spectra and fragmentation patterns. Among the regioisomers of 2 tested (5–8), only carveol (5) showed a significant response with either wild-type CymR or S77L. However, it will be distinguished during P450 mutant profiling by GC-MS, based on its unique retention time and characteristic mass fragmentation pattern. Since the intention was to develop a biosensor platform for screening 2-production, any mutant production strain identified by this biosensor platform will be followed up by searching for over-oxidized (3 and 4) products. Overall, these data indicate that S77L broadens CymR's responsiveness in a selective manner, with the strongest activation by the target product 2, supporting its use as a practical biosensor for screening hydroxylation pathways.

### CymR S77L-guided directed evolution of CYP153A6 for enhanced 2-production in *E. coli*

**Fig. 4:**
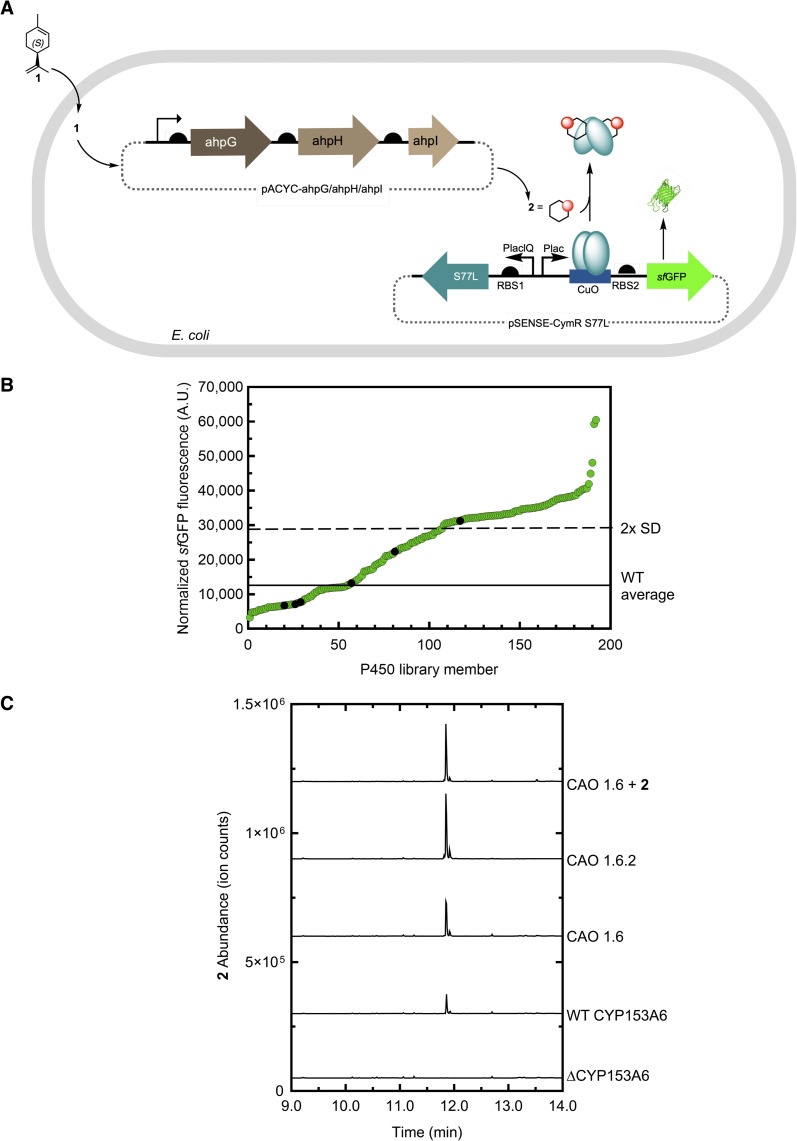
Biosynthesis and detection of 2 in an engineered *E. coli* strain. (A) Scheme showing the four-enzyme pACYC vector consisting of: CYP153A6 (ahpG), FdR (ahpH), and Fd (ahpI) for the hydroxylation of 1. Production of 2 is monitored by the engineered CymR-S77L biosensor (bottom plasmid). (B) Biosensor fluorescence responses of a representative portion of the CYP153A6 error-prone PCR library. Each point represents an individual clone ranked by normalized fluorescence signal. Wild-type CYP153A6 is shown in black. Variants exceeding the statistical cutoff were selected for secondary screening and GC-MS validation. (C) Extracted ion count (EIC) chromatograms of 2 (*m*/*z* = 152) determined by GC-MS analysis of the extracted culture media. Error bars represent the standard error of the mean (*n* = 3).

The wild-type CYP153A6 was co-expressed with its previously reported redox partners, ferredoxin reductase (FdR, AhpH) and ferredoxin (Fd, AhpGI), in *E. coli* BL21(DE3) that also harbored the pSENSE2-CymR-S77L biosensor plasmid (Fig. 4a). Consistent with previous studies,^27,32^ this strain supported the *in situ* production of 2 from 1, albeit at low levels (∼50 mg L^−1^ culture) as judged by gas chromatography-mass spectrometry (GC-MS) analysis (SI Fig. S3), but here with the ability of biosensor detection for the first time (Fig. 4b).

**Fig. 5:**
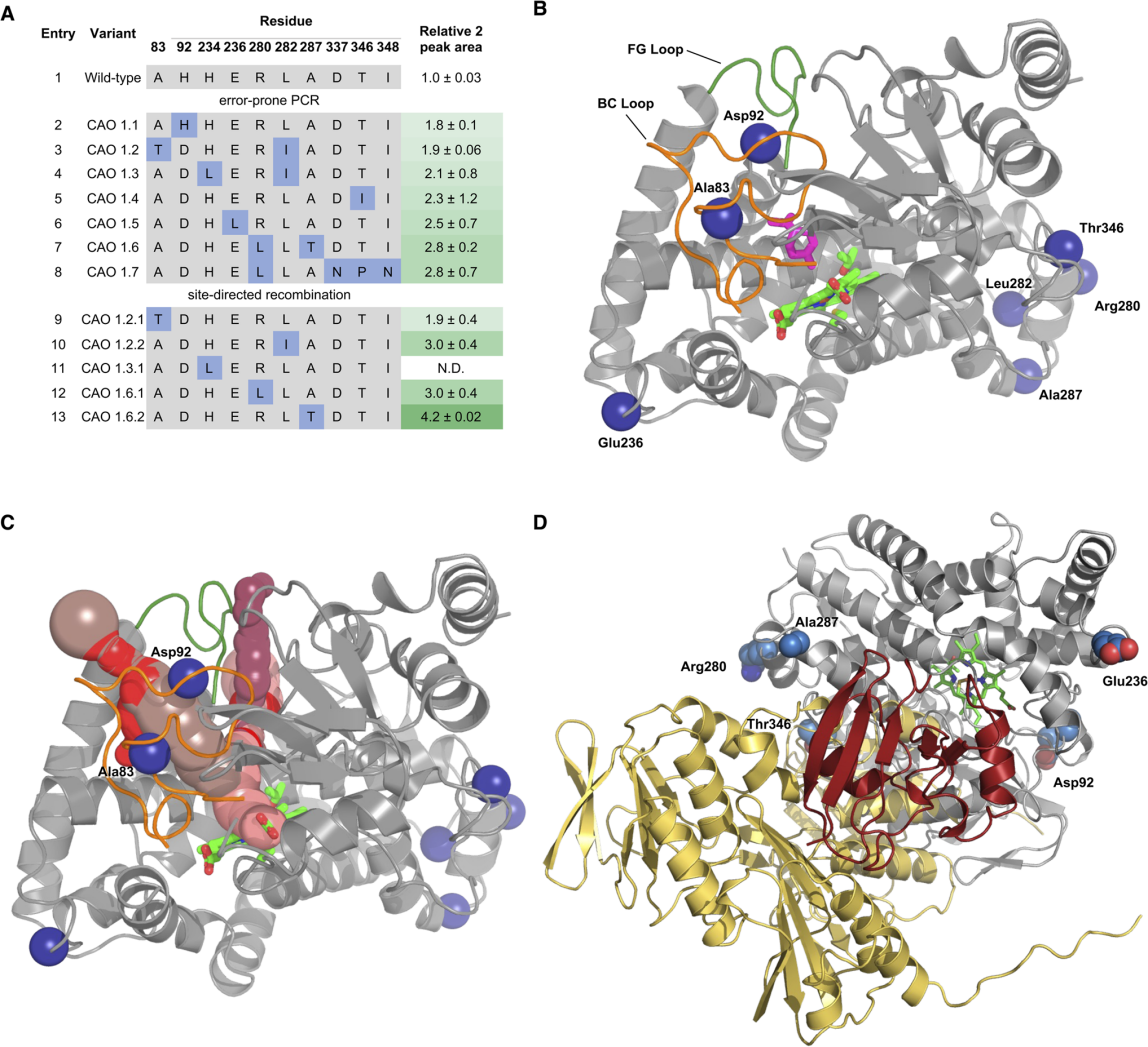
Sequence analysis and structural mapping of CYP153A6 variants identified *via* biosensor-guided screening. (A) Heatmap sequence alignment of selected hit variants, with CYP153A6 wild-type residues shown in gray and substitutions shown in blue. Relative fold improvements in product formation, determined by GC-MS analysis, are summarized to the right. (B) Model of CYP153A6 highlighting sites of confirmed functional mutations in the top confirmed hits (shown as spheres). 1 is shown as magenta sticks. Heme is shown as green sticks. Cys367 and Thr260 are shown as green sticks below and above the heme, respectively. The BC and FG loops are labeled. (C) Model of CYP153A6 with predicted substrate channels shown in shades of red. (D) Predicted CYP163A6:FdR:Fdx complex structure. FdR is shown in yellow. Fdx is shown in red. Selected residues are highlighted as spheres.

To address the likely bottleneck associated with CYP153A6, error-prone PCR was used to construct a library of randomly mutated P450 variants, with an average of 1–2 amino acid changes per gene product. The CYP153A6 random mutant library was co-transformed with the CymR S77L biosensor into *E. coli* BL21(DE3) for high-throughput screening in microplates. A total of ∼1500 library members were screened, and variants with fluorescence exceeding two standard deviations above the population average were classified as preliminary hits and advanced to secondary screening (Fig. 4b). The most responsive clones were regrown and subjected to GC-MS analysis to confirm increased 2 production levels. The resulting seven variants (CAO 1.1–1.7) were carried forward for further characterization in the absence of the biosensor plasmid. The production of 2 by each variant was determined using GC-MS analysis of the culture extracts. Notably, 2-production was not observed in the control strain lacking the P450 enzyme (ΔP450) (Fig. 4c). However, the CYP153A6 variants produced mass ions consistent with 2 that co-eluted with an authentic commercial standard (Fig. 4c). The most active variants (CAO 1.6/1.7) supported almost 3-fold higher 2-production than the wild-type CYP153A6 (Fig. 4c and 5a).

The DNA sequences of the seven P450 variants with improved 2 production revealed three single, three double, and one quadruple mutant: D92H (CAO 1.1), A83T/L282I (CAO 1.2), H234L/L282I (CAO 1.3), T346I (CAO 1.4), E236L (CAO 1.5), R280L/A287T (CAO 1.6), and L282I/D337N/T346P/I348N (CAO 1.7) (Fig. 5a).

### Site-directed recombination of beneficial mutations

Given that many hits include multiple mutations, the next round of directed evolution focused on deconvoluting the top variants from the library using site-directed mutagenesis to further improve activity. Five variants were rationally reconstructed and evaluated with the aim of (i) determining which substitutions were deleterious, (ii) identifying dominant mutations that improved activity in combination with others, and (iii) establishing which single mutations were sufficient to enhance production independently. The variants were evaluated for product formation by GC-MS analysis (Fig. 5a).

Variant CAO 1.2 was deconvoluted into its two constituent single mutants, A83T (CAO 1.2.1) and L282I (CAO 1.2.2). A83T retained nearly double the activity of the wild-type strain, whereas L282I exhibited 3-fold and 1.5-fold higher conversion compared to the wild-type and parent CAO 1.2, respectively (Fig. 5a). These findings indicate that the Thr substitution at position 83 may be mildly detrimental in the context of CAO 1.2 and that L282I could be combined with other beneficial single mutations to enhance activity further. Intriguingly, CAO 1.3 also shares the mutation L282I. The other contributing mutant in CAO 1.3, H234L, was completely inactive (CAO 1.3.1, entry 11, Fig. 5a), indicating that L282I is the sole beneficial mutation in both CAO 1.2 and CAO 1.3. Together, these observations demonstrate the overall significance of L282I.

CAO 1.6 was the joint-most active mutant but contained only two mutations compared to the wild-type, whereas CAO 1.7 contained four. Accordingly, the two contributing single mutants in CAO 1.6 (R280L and A287T) were constructed and characterized. Interestingly, both single mutants were more active than the wild-type, with A287T (CAO 1.6.2) showing the most activity of any variant characterized so far, with a 4.2-fold improvement over the wild type (Fig. 5a). Thus, although each mutation is beneficial, their combination in CAO 1.6 is somewhat detrimental. These results suggest that substituting threonine at position 287 provides a major structural benefit that is not further enhanced and may even be somewhat reduced when combined with the R280L mutation. Importantly, A287T emerged as the most consistently impactful single substitution across the panel, reinforcing its role as the principal driver of enhanced CYP153A6 activity in this system.

Interestingly, all confirmed beneficial mutations are located (Ala83, Asp92, Glu236, Arg280, Leu282, Ala287, Thr346) on the surface of the CYP153A6 protein, as judged by analysis of the CYP153A6 model. In fact, all beneficial mutations, except R280L, are located in loop regions (Fig. 5b). Ala83 and Asp92 are in the B/C loop, a region often associated with substrate binding in the CYP153 family.^65,66^ These two residues line one of the predicted substrate channels (Fig. 5c). Computational prediction of the CYP153A6 ternary complex with the FdR and Fd suggests that two of the beneficial mutations (R280L, A287T) are close to the CYP153A6:FdR interface and could influence the interaction. Consistent with the prediction that most of the beneficial mutations play a role in substrate channeling or redox partner interactions, the expression levels of the CYP153A6 variants are not significantly different than the wild-type enzyme, as judged by SDS-PAGE analysis of crude extracts (SI Fig. S4).

### Product specificity of wild-type and variant CYP153A6

A series of other potential 2 regioisomers and over-oxidized derivatives (2–11, Fig. 3a) all display unique GC-MS retention times (SI Fig. S5). However, only 2 was a detectable regioisomeric product (*m*/*z* 152) of the engineered CYP153A6 variants, as determined by GC-MS analysis (Fig. 4c), and mass ions for over-oxidized derivatives were not detected (data not shown). Subsequently, the regio- and chemo-selectivity of the wild-type enzyme has been maintained in the engineered CYP153A6 variant CAO 1.7.

## Conclusion

This study shows how combining biosensor-based high-throughput screening with targeted mutagenesis can enhance P450 enzyme performance, providing a powerful complement to rational and computational approaches to engineering P450s.^67,68^ By coupling enzymatic activity directly to a genetic readout, this platform enabled rapid identification of productive variants from mutant libraries. Through systematic mutagenesis, recombination, and analysis of beneficial mutations, we identified a single amino acid variant (A287T) that increased productivity by 4.2-fold compared to the wild-type CYP153A6. Importantly, without specifically screening the product selectivity of the library members, these improvements preserved selectivity for the target hydroxylated product. Overall, these results demonstrate the power of targeted, biosensor-guided enzyme engineering to yield significant gains in activity, laying a strong foundation for future rounds of combinatorial evolution, structure–function analysis, and pathway-level optimization in this and similar biocatalyst systems. Indeed, the broad specificity of the engineered CymR S77L biosensor and that of the recently reported CymR-based biosensor (F107S/R168E/V172S) for α-terpineol^52^ suggest that biosensors for a variety of monoterpenes and their modifications could be developed. Inspired by the development of selective transcription factor biosensors for other compound classes^44,45,50,69^ and based on the modest structural and chemical differences between 1 and 2, and the relative ease of acquiring effector selectivity, we predict that other biosensors could be developed to report on a variety of natural and abiotic terpene modifications.

## Author contributions

A. M. and G. J. W. initially conceived the project, with subsequent contributions from C. A. O. A. M. conducted the CymR-directed evolution and preliminary CYP153A6 directed evolution that informed the campaign described here. C. A. O. conducted the CYP153 engineering that led to the identification and characterization of the CYP153A6 variants C. A. O 1.1–1.7 and derivatives. The CymR data was originally analyzed by A. M. and G. J. W., while the CYP153A6 variant data was analyzed by C. A. O. and G. J. W. The manuscript was written by C. A. O. and G. J. W., with contributions and proofreading from all authors.

## Conflicts of interest

There are no conflicts to declare.

## Supplementary Material

RA-OLF-D6RA03129C-s001

## Data Availability

The data supporting this article have been included as part of the supplementary information (SI). Raw data files are freely available upon request. Supplementary information: Tables S1–S2, SI Fig. S1–S8, and experimental details. See DOI: https://doi.org/10.1039/d6ra03129c.
