# Supplementary material for: Directed evolution of a cytochrome P450 monooxygenase for improved perillyl alcohol biosynthesis via a tailored genetically encoded biosensor
Source: RSC Adv. 2026 Jul 3;16(35):35590–600. doi: 10.1039/d6ra03129c (PMC13329868; doi:10.1039/d6ra03129c)
Supplement: RA-016-D6RA03129C-s001 [file RA-016-D6RA03129C-s001.pdf]

Supplementary Information (SI) for RSC Advances.
This journal is © The Royal Society of Chemistry 2026

Directed evolution of a cytochrome P450 monooxygenase for improved perillyl alcohol biosynthesis via a tailored genetically encoded biosensor

Catherine A. Odhiambo[1†], Alexandra A. Malico[1,2†], and Gavin J. Williams[1,3*]

[1] Department of Chemistry, NC State University, Raleigh, North Carolina 27695, United States

[2] Present address: BASF Corporation, Florham Park, New Jersey 07932, United States

[3] Comparative Medicine Institute, NC State University, Raleigh, North Carolina 27695, United States

[†] These authors contributed equally

* Corresponding author, gjwillia@ncsu.edu

Table of Contents

Supplementary Tables

Supplemental Table S1. Oligonucleotides used in this study.
Supplemental Table S2. Plasmid sequences.

Supplemental Figures

Supplementary Figure S1. The plasmid map of pSENSE3-CymR (top) and pACYCDuet-P450-RED-Fd (bottom).
Supplementary Figure S2. Wild-type and variant S77L CymR biosensor strain detection of **1**/**2** mixtures simulating conversion of **1** to **2** in vivo.
Supplementary Figure S3. GC–MS identification and quantification of **2** from culture extracts.
Supplementary Figure S4. SDS-PAGE whole cell lysate analysis of wild-type and mutant **2** production strains.
Supplementary Figure S5. GC-MS profiles of hydroxylated **1**-derivatives.

Supplemental Methods

Construction of pSENSE-CymR biosensor circuit plasmid
Structure-guided saturation mutagenesis of CymR
Construction of the prototype 2-producing *E. coli* strain
Error-prone PCR of wild-type CYP153A6
Site-directed mutagenesis of CYP153A6

Supplementary Tables

Supplemental Table S1. Oligonucleotides used in this study.

Primer #	Name	Nucleotide Sequence	Function
Construction of pSENSE-CymR biosensor			
1	cuO-F	GTTTCCTCCTGTTAGCagaaacaaaccaacctgtctgtattaTTATATTCAATCCCACAC	
2	cuO-R	GTGTGGGATTGAATATAAtaatacagacaggttggtttgtttctGCTAACAGGAGGAAAC	cloning the operator sequence (cuO)
3	cym-F	ataCATATGTCCCCTAAGC	
4	cym-R	ataGGTACCCTACCG	amplification of *CymR*
Saturation mutagenesis of CymR			
5	S77X-F	ATTACGGAACGGNNKCGGGCAAGACTGGCT	
6	S77X-R	AGCCAGTCTTGCCCGMNNCCGTTCCGTAAT	saturation library of Ser77
7	Y70X-F	TTCGAATGGCTGNNKGAACAGATTACGGAA	
8	Y70X-R	TTCCGTAATCTGTTCMNNCAGCCATTCGAA	saturation library of Tyr70
9	F102X-F	GCCGCCGAATTTNNKCTTGATGATGACTTT	
10	F102X-R	AAAGTCATCATCAAGMNNAAATTCGGCGGC	Saturation library Phe192
11	S110X-F	GATGACTTTTCCATANNKCTTGATCTTATAGTT	
12	S110X-R	AACTATAAGATCAAGMNNTATGGAAAAGTCATC	Saturation library of ser110
13	N134X-F	AGAACGGTTGAACGTNNKCGGTTCGTGGTTGAA	
14	N134X-R	TTCAACCACGAACCGMNNACGTTCAACCGTTCT	Saturation library of Asn134
Error prone PCR of P450			
15	RED/Fd-F	gcgtaaggatctagGACGTCaaagaaatgatacatacaggcg	
16	RED/Fd-R	taaacaaaattattGGATCCggggaattgttatccgctca	linearizing pACYC-RED/Fd for P450
17	P450-F	tgagcggataacaattccccGGATCCaataattttgtttaactttaataa	
18	P450-R	TcctgtatgtatcatttcttttGACGTCctagatccttacgc	amplification of *P450*
Site directed mutagenesis of P450 variants (deconvolution of p450 hits)			
19	83T-F	TTCAAGCGAGactAAATCAGGTG	
20	83T-R	AATACTTTCGGGTTCGTTTC	Thr at Ala83
21	92H-F	TACCATAATGcatGATAACGCCG	
22	92H-R	ATACCACCTGATTTAGTC	His at Asp92
23	234L-F	TATGATGGCActgTCGGAGAGTAC	

24	234L-R	GAGATCAGATCATTTTTTGG	Leu at His234
25	280L-F	CGATGAATATctgAAGCTAAGTGC	
26	280L-R	GGAAATTCGTTTAGGGCAAG	Leu at Arg280
27	282I-F	ATATCGGAAGataAGTGCGAATC	
28	282I-R	TCATCGGGAAATTCGTTTAG	Ile at Leu282
29	287T-F	TGCGAATCCTacaTTGATCTCGTC	
30	287T-R	CTTAGCTTCCGATATTCATCG	Thr at Ala287
31	337N-F	CGGCAATCGAaacCCAGAAGCTA	
32	337N-R	GAAACATACCACATAACCACC	Asn at Asp337
33	348N-F	TGATACATTTaacATCGATCGTGC	
34	348N-R	GGGTTGTCGATAGCTTC	Asn at Ile348
Construction of M1 controls			
35	P450Tr-F	GCTGCTCATCtaaGGAGGGAATG	
36	P450Tr-R	ACGATGTTCCCTAAGTATTC	Stop codon at Val255
37	Tr29A-F	TAGCAATCCGgctCTGTTCCGCG	
38	Tr29A-R	ACATCAATATCTTCCAGGGCCATG	Ala at Val29
39	RED-P123T-F	TTTAGCCTGTaccGGAGCGGACC	
40	RED-P123T -R	GGTCTGGGCCTCCCCCCG	Thr at Pro123
41	RED-W327C-F	TCCCTGGTTCtgcTCGGATCAGT	
42	RED-W327C-R	ACCTGACGAAACGGTTCAT	Trp at Cys327

Supplemental Table S2. Plasmid sequences.

Plasmid Name	Sequence (5'-3')
pSENSE-CymR/*sf*GFP<br>CymR (**purple**)<br>NCBI: U24215.1<br>CuO (**red**)<br>*sf*GFP (**green**)<br>NCBI: MK301203.1	TTGAGATCCTTTTTTTCTGCGCGTAATCTGCTGCTTGCAAACAAAAAAACCACCGCTACCAGCGGTGGTTTGTTTGCCGGATCAAGAGCTACCAACTCT<br>TTTTCCGAAGGTAACTGGCTTCAGCAGAGCGCAGATACCAAATACTGTCCTTCTAGTGTAGCCGTAGTTAGGCCACCACTTCAAGAACTCTGTAGCACC<br>GCCTACATACCTCGCTCTGCTAATCCTGTTACCAGTGGCTGCTGCCAGTGGCGATAAGTCGTGTCTTACCGGGTTGGACTCAAGACGATAGTTACCGGA<br>TAAGGCGCAGCGGTCGGGCTGAACGGGGGGTTCGTGCACACAGCCCAGCTTGGAGCGAACGACCTACACCGAACTGAGATACCTACAGCGTGAGCTATG<br>AGAAAGCGCCACGCTTCCCGAAGGGAGAAAGGCGGACAGGTATCCGGTAAGCGGCAGGGTCGGAACAGGAGAGCGCACGAGGGAGCTTCCAGGGGGAAA<br>CGCCTGGTATCTTTATAGTCCTGTCGGGTTTCGCCACCTCTGACTTGAGCGTCGATTTTTGTGATGCTCGTCAGGGGGGCGGAGCCTATGGAAAAACGC<br>CAGCAACGCGGCCTTTTTACGGTTCCTGGCCTTTTGCTGGCCTTTTGCTCACATGTTCTTTCCTGCGTTATCCCCTGATTCTGTGGATAACCGTATTAC<br>CTCTAGTGTACAGTGATCAAGACTTCGATACCACCGACCGTACCGGTACTAATCGACGACGGTCGTGTTCGTCGCCTGCCGCAGGGACTCTGCACACCT<br>TAAGCTATTTGTACAACTCATCCATCCCTCCAGTTGAGTGGCGTCCTTCGGCCCGCTCATACTGTTCTACAATTGTATAGTCTTCGTTATGGGACGTGA<br>TGTCCAACTTAATATTAACATTATAGGCACCGGGAAGCTGCACGGGTTTTTTAGCCTTGTAAGTAGTTTTGACCTCAGCATCGTAGTGTCCACCGTCTT<br>TTAATTTCAGACGCTGTTTGATCTCCCCCTTTAACGCCCCATCTTCTGGGTACATACGTTCGCTCGATGCTTCCCATCCCATAGTCTTTTTCTGCATGA<br>CAGGGCCGTCTGACGGGAAATTGGTCCCCCGAAGTTTCACTTTATATATGAACTCCCCATCCTGAAGGGAGGAGTCCTGGGTCACAGTGACCACTCCGC<br>CATCTTCGAAGTTCATCACGCGCTCCCACTTGAACCCCTCGGGGAACGATAATTTAAGGTAGTCTGGTATGTCGGCTGGATGCTTGACGTACGCTTTAC<br>TCCCATACATAAACTGCGGAGAAAGAATGTCCCACGCAAAAGGAAGCGGACCACCTTTAGTAACTTTTAACTTTGCCGTCTGAGTCCCCTCGTACGGGC<br>GACCCTCGCCCTCACCCTCGATTTCAAACTCGTGGCCGTTAACGCTACCCTCCATGTGTACCTTGAATCTCATAAACTCCTTGATGATCGCCATATTGT<br>CCTCTTCGCCCTTGCTGACCATGAATTCTTAGCCTTATTTTGGATACTATGGGTACCCTA**CCGTTTAAATTTGGCGTAGCGTTCGCGAGCAATTTCAAG**<br>**GGTTGAGTTACGGACTCTTTCGAAGCGTTCTTTGTCCTTCTGCCATAAAGAACGCACTGCAAGGCCCCGTACTGAGTTGAATATCAACCATAATATGTC**<br>**CTCGGCATCATCACGTGACAGTCCCCGCGAGACTAATACACCAAGCCACATGTCTTCAACCACGAACCGGTTACGTTCAACCGTTCTCTGAATACCTTC**<br>**CCGAAGAGCTGGATC**ACGATCCGCGGCAACTATAAGATCAAGGGATATGGAAAAGTCATCATCAAGGAAAAATTCGGCGGCGTCGTCCAACATTTGCTG<br>GATCACGTCATCTTCCGGCTTCAATTTAGCCAGTCTTGCCCGACTCCGTTCCGTAATCTGTTCGTACAGCCATTCGAAGGTAGCCAGCAACAGTTCCAA<br>CTTAGTAGGGAAATGGTGACTCTGAGCACCCCGGCTGACTCCCGCGGCCCCAGGAACGTCAGCAATTCTGAAGCCCGCGTATCCCTTCTCGCGTAAAAC<br>TCCAAGTGCAGCCGCTATCAATTTACCTTGCGTCTCCATAGCACGCTCCGCTTGGGTCCGGCGCTTAGGGGACATATGGCAACCTCCTTGCCGCGGAGT<br>GGAGGATCGTTGGGCGCTATCATGCCATACCGCGAAAGGTTTTGCACCATCTAGAGCGCAACGCAAT**TAATGTGAGTTAGCTCACTCAT**TAGGCACCCC<br>AGGCTTTACACTTTATGCTTCCGGCTCGTATGTTGCCTAGGAACAAACAGACAATCTGGTCTGTTTGTAAACCTAAAAGAAAAAAGAGAGGAGGCATAT<br>TACTAGGACTATTAGTACCTAGTCTTAATTGTCCGGCATCCGAAACAGCTAAGGAGTTACCTAGT**ATGAGCAAAGGAGAAGAACTTTTCACTGGAGTTG**<br>**TCCCAATTCTTGTTGAATTAGATGGTGATGTTAATGGGCACAAATTTTCTGTCCGTGGAGAGGGTGAAGGTGATGCTACAAACGGAAAACTCACCCTTA**<br>**AATTTATTTGCACTACTGGAAAACTACCTGTTCCGTGGCCAACACTTGTCACTACTCTGACCTATGGTGTTCAATGCTTTTCCCGTTATCCGGATCACA**<br>**TGAAACGGCATGACTTTTTCAAG**AGTGCCATGCCCGAAGGTTATGTACAGGAACGCACTATATCTTTCAAAGATGACGGGACCTACAAGACGCGTGCTG<br>AAGTCAAGTTTGAAGGTGATACCCTTGTTAATCGTATCGAGTTAAAGGGTATTGATTTTAAAGAAGATGGAAACATTCTTGGACACAAACTCGAGTACA<br>ACTTTAACTCACACAATGTATACATCACGGCAGACAAACAAAAGAATGGAATCAAAGCTAACTTCAAAATTCGCCACAACGTTGAAGATGGTTCCGTTC<br>AACTAGCAGACCATTATCAACAAAATACTCCAATTGGCGATGGCCCTGTCCTTTTACCAGACAACCATTACCTGTCGACACAATCTGTCCTTTCGAAAG<br>ATCCCAACGAAAAGCGTGACCACATGGTCCTTCTTGAGTTTGTAACTGCTGCTGGGATTACACATGGCATGGATGAGCTCTACAAAAAGCTTGGGCCCG<br>TTTAAACGGTCTCCAGCTTGGCTGTTTTGGCGGATGAGAGAAGATTTTCAGCCTGATACAGATTAAATCAGAACGCAGAAGCGGTCTGATAAAACAGAA<br>TTTGCCTGGCGGCAGTAGCGCGGTGGTCCCACCTGACCCCATGCCGAACTCAGAAGTGAAACGCCGTAGCGCCGATGGTAGTGTGGGGTCTCCCCATGC<br>GAGAGTAGGGAACTGCCAGGCATCAAATAAAACGAAAGGCTCAGTCGAAAGACTGGGCCTTTCGTTTTATCTGTTGTTTGTCGGTGAACGCTCTCCTGA<br>GTAGGACAAATCCGCCGGGAGCGGATTTGAACGTTGCGAAGCAACGGCCCGGAGGGTGGCGGGCAGGACGCCCGCCATAAACTGCCAGGCATCAAATTA<br>AGCAGAAGGCCATCCTGACGGATGGCCTTTTTGCGTTTCTACAAACGCATGCTCTTTTTGTTTATTTTTCTAAATACATTCAAATATGTATCCGCTCAT<br>GAGACAATAACCCTGATAAATGCTTCAATAATATTGAAAAAGGAAGAGTATGAGTATTCAACATTTCCGTGTCGCCCTTATTCCCTTTTTTGCGGCATT<br>TTGCCTTCCTGTTTTTGCTCACCCAGAAACGCTGGTGAAAGTAAAAGATGCTGAAGATCAGTTGGGTGCACGAGTGGGTTACATCGAACTGGATCTCAA<br>CAGCGGTAAGATCCTTGAGAGTTTTCGCCCCGAAGAACGTTTTCCAATGATGAGCACTTTTAAAGTTCTGCTATGTGGCGCGGTATTATCCCGTGTTGA<br>CGCCGGGCAAGAGCAACTCGGTCGCCGCATACACTATTCTCAGAATGACTTGGTTGAGTACTCACCAGTCACAGAAAAGCATCTTACGGATGGCATGAC<br>AGTAAGAGAATTATGCAGTGCTGCCATAACCATGAGTGATAACACTGCGGCCAACTTACTTCTGACAACGATCGGAGGACCGAAGGAGCTAACCGCTTT<br>TTTGCACAACATGGGGGATCATGTAACTCGCCTTGATCGTTGGGAACCGGAGCTGAATGAAGCCATACCAAACGACGAGCGTGACACCACGATGCCTGT<br>AGCAATGGCAACAACGTTGCGCAAACTATTAACTGGCGAACTACTTACTCTAGCTTCCCGGCAACAATTAATAGACTGGATGGAGGCGGATAAAGTTGC<br>AGGACCACTTCTGCGCTCGGCCCTTCCGGCTGGCTGGTTTATTGCTGATAAATCTGGAGCCGGTGAGCGTGGGTCTCGCGGTATCATTGCAGCACTGGG<br>GCCAGATGGTAAGCCCTCCCGTATCGTAGTTATCTACACGACGGGGAGTCAGGCAACTATGGATGAACGAAATAGACAGATCGCTGAGATAGGTGCCTC<br>ACTGATTAAGCATTGGTAACTGTCAGACCAAGTTTACTCATATCCATGGATACTTTAGATTGATTTAAAACTTCATTTTTAATTTAAAAGGATCTAGGT<br>GAAGATCCTTTTTGATAATCTCATGACCAAAATCCCTTAACGTGAGTTTTCGTTCCACTGAGCGTCAGACCCCGTAGAAAAGATCAAAGGATCTTC

pACYCDuet-P450/RED/Fd<br><br>P450(**blue**)<br>(original sequence, GenBank: AJ783967.1)<br><br>RED (**yellow**)<br>(original sequence, GenBank: AFO66438.1)<br><br>Fd (**maroon**)<br>(original sequence, NCBI: WP_099250445.1)	CCGCCGCTTTACAGGCTTCGACGCCGCTTCGTTCTACCATCGACACCACCACGCTGGCACCCAGTTGATCGGCGCGAGATTTAATCGCCGCGACAATT<br>TGCGACGGCGCGTGCAGGGCCAGACTGGAGGTGGCAACGCCAATCAGCAACGACTGTTTGCCCGCCAGTTGTTGTGCCACGCGGTTGGGAATGTAATT<br>CAGCTCCGCCATCGCCGCTTCCACTTTTTCCCGCGTTTTCGCAGAAACGTGGCTGGCCTGGTTCACCACGCGGGAAACGGTCTGATAAGAGACACCGG<br>CATACTCTGCGACATCGTATAACGTTACTGGTTTCACATTCACCACCCTGAATTGACTCTCTTCCGGGCGCTATCATGCCATACCGCGAAAGGTTTTG<br>CGCCATTCGATGGTGTCCGGGATCTCGACGCTCTCCCTTATGCGACTCCTGCATTAGGAAATTAATACGACTCACTATAGGGGAATTGTGAGCGGATA<br>ACAATTCCCCGGATCCAATAATTTTGTTTAACTTTAATAAGGAGATATAC**ATGACTGAAATGACTGTCGCGGCTTCGGACGCAACTAATGCCGCCTAC**<br>**GGCATGGCCCTGGAAGATATTGATGTTAGCAATCCGGTTCTGTTCCGCGATAATACGTGGCACCCCTATTTCAAAAGGCTGCGTGAGGAAGACCCAGT**<br>**GCACTATTGTAAGTCCTCTATGTTTGGTCCATACTGGAGTGTTACCAAATATCGTGATATAATGGCCGTGGAAACGAACCCGAAAGTATTTTCAAGCG**<br>**AGGCTAAATCA**GGTGGTATTACCATAATGGATGATAACGCCGCTGCTAGCCTGCCTATGTTTATCGCGATGGACCCCCCGAAACATGATGTTCAGCGA<br>AAAACCGTATCCCCTATTGTTGCGCCGGAGAATTTAGCCACGATGGAGTCAGTCATACGCCAGAGAACGGCTGACTTGCTTGATGGTTTACCCATAAA<br>TGAAGAATTTGACTGGGTTCATCGCGTCTCCATTGAGTTAACCACCAAAATGCTGGCGACCCTGTTTGACTTCCCCTGGGACGATAGAGCGAAATTGA<br>CTCGCTGGTCCGATGTGACCACAGCTTTACCCGGAGGTGGTATAATTGATAGCGAAGAACAGCGTATGGCCGAGTTGATGGAGTGCGCTACGTACTTC<br>ACCGAACTGTGGAATCAGCGTGTTAACGCTGAGCCAAAAAATGATCTGATCTCTATGATGGCACACTCGGAGAGTACTAGACATATGGCTCCGGAGGA<br>ATACTTAGGGAACATCGTGCTGCTCATCGTAGGAGGGAATGATACGACCCGTAACTCCATGACGGGTGGTGTACTTGCCCTAAACGAATTTCCCGATG<br>AATATCGGAAGCTAAGTGCGAATCCTGCATTGATCTCGTCAATGGTGTCCGAAATCATTCGGTGGCAGACCCCTCTCTCCCATATGCGCCGGACGGCT<br>CTTGAAGACATAGAGTTTGGTGGAAAACATATTAGACAAGGAGACAAGGTGGTTATGTGGTATGTTTCCGGCAATCGAGATCCAGAAGCTATCGACAA<br>CCCTGATACATTTATTATCGATCGTGCAAAGCCACGTCAACACCTGTCTTTTGGCTTCGGTATTCATCGTTGCGTTGGTAATCGCCTGGCGGAATTAC<br>AATTAAATATTCTGTGGGAAGAGATTCTGAAACGATGGCCAGACCCCCTTCAGATTCAAGTTTTACAGGAACCAACGCGTGTTCTGAGTCCTTTTGTA<br>AAAGGTTATGAATCGCTGCCAGTACGCATCAACGCGTAAGGATCTAGGACGTCAAAGAA**ATGATACATACAGGCGTCACGGAGGCTGTTGTGGTCGTC**<br>**GGTGCTGGGCAGGCGGGAGCACAGACTGTGACAAGTTTGCGACAGCGCGGTTTTGAAGGTCAGATTACGCTGTTAGGAGATGAACCCGCGTTACCGTA**<br>**TCAGCGCCCACCGTTATCCAAAGCATTTTTGGCTGGCACACTGCCACTGGATCGCCTGTATTTACGCCCAGCCGCGTTTTACCAACAGGCACATGTGG**<br>**ATGTCATGGTCGATACAGGT**GTCTCGGAACTAGATACCGAAAATCGTCGTATCCGGCTGACAGATGGACGAGCGATATCTTTTGACCATCTGGTTTTA<br>GCCACCGGGGGGAGGCCCAGACCTTTAGCCTGTCCCGGAGCGGACCACCCCCGCGTTCATTATTTGCGTACAGTCACCGATGTTGATCGTATTCGGTC<br>ACAGTTTCATCCTGGCACCCGACTTGTATTAGTGGGGGGGGGGTACATAGGGTTAGAAATTGCAGCGGTAGCCGCCGAATTAGGTTTGACTGTAACAG<br>TGCTTGAGGCCCAGACAACTGTGTTGGCCCGCGTTACGTGTCCAACCGTCGCACGCTTTTTCGAGCATACACACCGGCGCGCGGGTGTCACCATTCGC<br>TGCGCCACGACCGTGACACGGATTCATGATTCTTCGAGTACTGCTCGAATCGAGCTCGACTCAGGGGAATATATTGACGCGGATCTGGTTATAGTGGG<br>TATTGGTTTGCTCCCCAACGTTGACCTTGCAAGCGCAGCTGGCCTCACGTGCGAAAGTGGCATTGTGGTAGACAGTCGTTGCCAAACGTCCGCACCTG<br>GAATCTACGCAGCCGGCGATTGCACGCAATATCCATCCCCTATCTACGGTCGGCCACTTCATTTGGAATCTGTTCATAATGCGATCGAACAGGCTAAG<br>ACCGCAGCAGCCGCAATCTTGGGGCGGGATGAACCGTTTCGTCAGGTTCCCTGGTTCTGGTCGGATCAGTACAACATTAAACTTCAGACCGCAGGCGT<br>TAATGAGGGTTACGATGATGTCATCATTCGCGGTGACCCCGCATCAGCGAGCTTTGCCGCATTTTACTTAAGAGCGGGGAAATTACTGGCTGTGGATG<br>CGATTAATCGGCCGCGTGAGTTCATGGCTTCCAAAACCCTGATTGCTGAAAGGGCCGAAGTAGACCCGACCCAATTGGCAGACGAGAGCTTGCCTCCG<br>ACCGCCCTGGCGGCAGCGGTCAATGGTCCTACTCGGGCTACGAGTCCCACAAGCTTATAAGGATCTAGGAGGATAAAGAA**ATGCCCAAGATTACGTAC**<br>**ATTGATTACACGGGAACGTCACGCTGTGTTGATGCTGAAAATGGTATGTCATTAATGGAAATTGCGATAAACAATAACGTTCCCGGAATTGATGGAGA**<br>**TTGCGGCGGCGAGTGCGCGTGTGCCACGTGTCATGTGCATGTTGACGCCGATTGGCTGGATAAATTACCTCCTTCCAGCGACCAGGAGGTTAGTATGT**<br>**TGGAATTTTGTGACGGTGTTGATCATACTTCCCGCTTAGGG**TGTCAGATTAAAATTTGCCCGACCTTAGATGGTATTGTGGTACGGACGCCCGCCGCG<br>CAGCACTAAGGATCCAAACTCGAGTAAGGATCTCCAGGCATCAAATAAAACGAAAGGCTCAGTCGAAAGACTGGGCCTTTCGTTTTATCTGTTGTTTG<br>TCGGTGAACGCTCTCTACTAGAGTCACACTGGCTCACCTTCGGGTGGGCCTTTCTGCGTTTATACCTAGGCTACAGCCGATAGTCTGGAACAGCGCAC<br>TTACGGTCAGGCATTTGAGAAGCACACGGGAACAGCGCACTTACGGTCAGGCATTTGAGAAGCACACGGTCACACTGCTTCCGGTAGTCAATAAACCG<br>GTAAACCAGCAATAGACATAAGCGGCTATTTAACGACCCTGCCCTGAACCGACGACCGGGTCGAATTTGCTTTCGAATTTCTGCCATTCATCCGCTTA<br>TTATCACTTATTCAGGCGTAGCAACCAGGCGTTTAAGGGCACCAATAACTGCCTTAAAAAAATTAGAAAAACTCATCGAGCATCAAATGAAACTGCAA<br>TTTATTCATATCAGGATTATCAATACCATATTTTTGAAAAAGCCGTTTCTGTAATGAAGGAGAAAACTCACCGAGGCAGTTCCATAGGATGGCAAGAT<br>CCTGGTATCGGTCTGCGATTCCGACTCGTCCAACATCAATACAACCTATTAATTTCCCCTCGTCAAAAATAAGGTTATCAAGTGAGAAATCACCATGA<br>GTGACGACTGAATCCGGTGAGAATGGCAAAAGTTTATGCATTTCTTTCCAGACTTGTTCAACAGGCCAGCCATTACGCTCGTCATCAAAATCACTCGC<br>ATCAACCAAACCGTTATTCATTCGTGATTGCGCCTGAGCGAGACGAAATACGCGATCGCTGTTAAAAGGACAATTACAAACAGGAATCGAATGCAACC<br>GGCGCAGGAACACTGCCAGCGCATCAACAATATTTTCACCTGAATCAGGATATTCTTCTAATACCTGGAATGCTGTTTTCCCGGGGATCGCAGTGGTG<br>AGTAACCATGCATCATCAGGAGTACGGATAAAATGCTTGATGGTCGGAAGAGGCATAAATTCCGTCAGCCAGTTTAGTCTGACCATCTCATCTGTAAC<br>ATCATTGGCAACGCTACCTTTGCCATGTTTCAGAAACAACTCTGGCGCATCGGGCTTCCCATACAATCGATAGATTGTCGCACCTGATTGCCCGACAT<br>TATCGCGAGCCCATTTATACCCATATAAATCAGCATCCATGTTGGAATTTAATCGCGGCCTAGAGCAAGACGTTTCCCGTTGAATATGGCTCATTTTA<br>GCTTCCTTAGCTCCTGAAAATCTCGATAACTCAAAAAATACGCCCGGTAGTGATCTTATTTCATTATGGTGAAAGTTGGAACCTCTTACGTGCCGATC<br>AACGTCTCATTTTCGCCAAAAGTTGGCCCAGGGCTTCCCGGTATCAACAGGGACACCAGGATTTATTTATTCTGCGAAGTGATCTTCCGTCACAGGTA<br>TTTATTCGGCGCAAAGTGCGTCGGGTGATGCTGCCAACTTACTGATTTAGTGTATGATGGTGTTTTTGAGGTGCTCCAGTGGCTTCTGTTTCTATCAG<br>CTGTCCCTCCTGTTCAGCTACTGACGGGGTGGTGCGTAACGGCAAAAGCACCGCCGGACATCAGCGCTAGCGGAGTGTATACTGGCTTACTATGTTGG<br>CACTGATGAGGGTGTCAGTGAAGTGCTTCATGTGGCAGGAGAAAAAAGGCTGCACCGGTGCGTCAGCAGAATATGTGATACAGGATATATTCCGCTTC<br>CTCGCTCACTGACTCGCTACGCTCGGTCGTTCGACTGCGGCGAGCGGAAATGGCTTACGAACGGGGCGGAGATTTCCTGGAAGATGCCAGGAAGATAC<br>TTAACAGGGAAGTGAGAGGGCCGCGGCAAAGCCGTTTTTCCATAGGCTCCGCCCCCCTGACAAGCATCACGAAATCTGACGCTCAAATCAGTGGTGGC<br>GAAACCCGACAGGACTATAAAGATACCAGGCGTTTCCCCCTGGCGGCTCCCTCGTGCGCTCTCCTGTTCCTGCCTTTCGGTTTACCGGTGTCATTCCG<br>CTGTTATGGCCGCGTTTGTCTCATTCCACGCCTGACACTCAGTTCCGGGTAGGCAGTTCGCTCCAAGCTGGACTGTATGCACGAACCCCCCGTTCAGT<br>CCGACCGCTGCGCCTTATCCGGTAACTATCGTCTTGAGTCCAACCCGGAAAGACATGCAAAAGCACCACTGGCAGCAGCCACTGGTAATTGATTTAGA<br>GGAGTTAGTCTTGAAGTCATGCGCCGGTTAAGGCTAAACTGAAAGGACAAGTTTTGGTGACTGCGCTCCTCCAAGCCAGTTACCTCGGTTCAAAGAGT<br>TGGTAGCTCAGAGAACCTTCGAAAAACCGCCCTGCAAGGCGGTTTTTTCGTTTTCAGAGCAAGAGATTACGCGCAGACCAAAACGATCTCAAGAAGAT<br>CATCTTATTAATCAGATAAAATATTTCTAGATTTCAGTGCAATTTATCTCTTCAAATGTAGCACCTGAAGTCAGCCCCATACGATATAAGTTGTAATT

Supplemental Figures

A

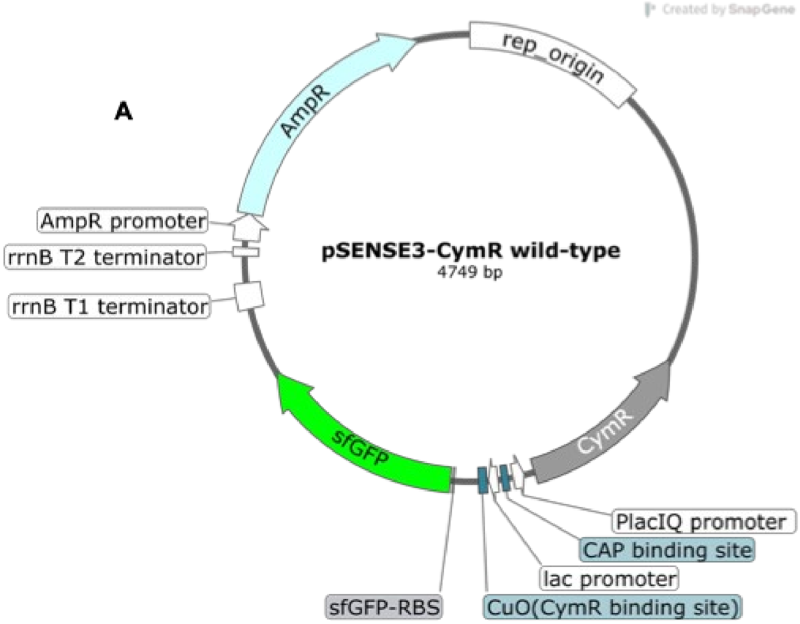

B

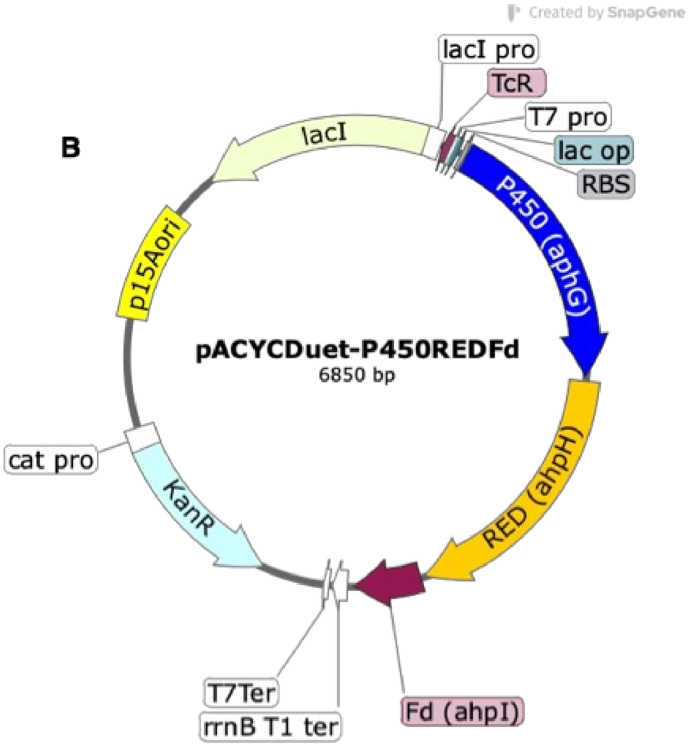

Supplementary Figure S1. The plasmid map of pSENSE3-CymR (top) and pACYCDuet-P450-RED-Fd (bottom).

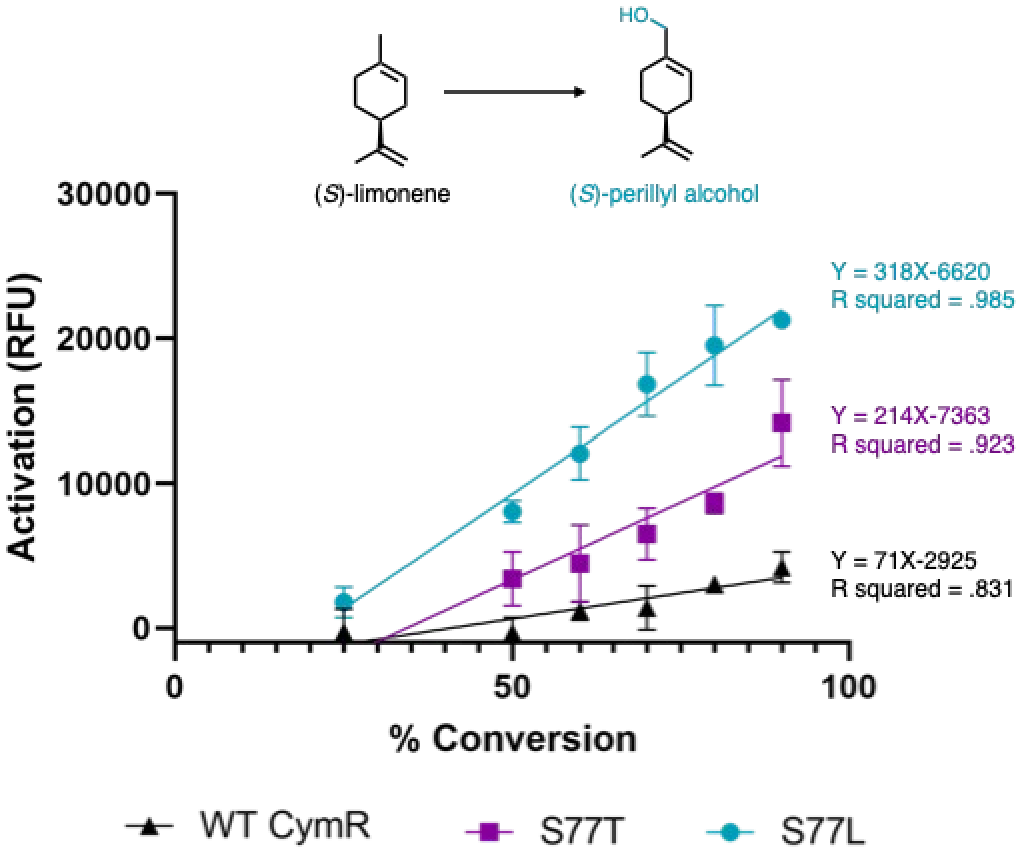

Supplementary Figure S2. Wild-type and variant S77L CymR biosensor strain detection of **1**/**2** mixtures simulating conversion of **1** to **2** in vivo. The data were fit to a simple linear regression. The total terpene concentration was 2.5 mM. Thus, 90% conversion is 2.25 mM **2** and 0.2 mM **1**. Error bars (where visible) represent standard deviation from the average (n = 3). S77T is an inferior CymR mutant not discussed in this manuscript.

A

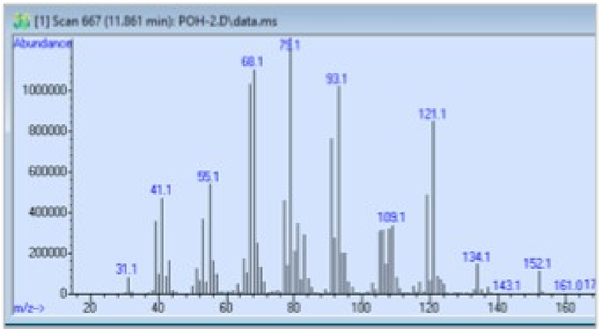

B

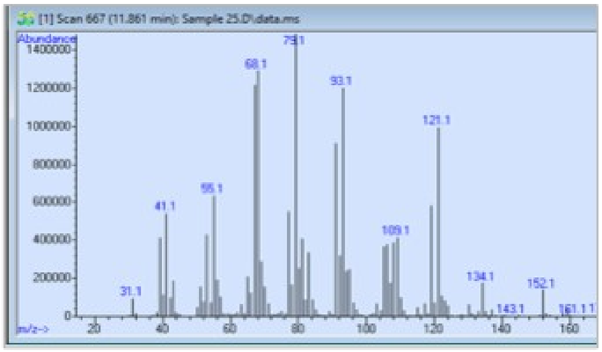

C

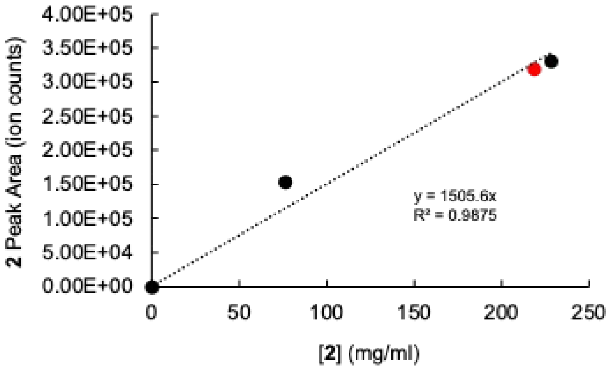

Supplementary Figure S3. GC–MS identification and quantification of **2** from culture extracts. **A**) Mass spectrum of **2** produced by CAO 1.6.2 showing characteristic fragment ions at *m*/*z* 68, 79, 121,134, and 152. **B**) Mass spectrum of commercial standard **2**. **C**) Calibration curve of **2** using EIC peak areas of commercial standard. CAO 1.6.2 (red) produced a concentration of **2** that was almost equal to one of the standard concentrations, estimated at ~219 mg/L in the culture supernatant according to the linear fit.

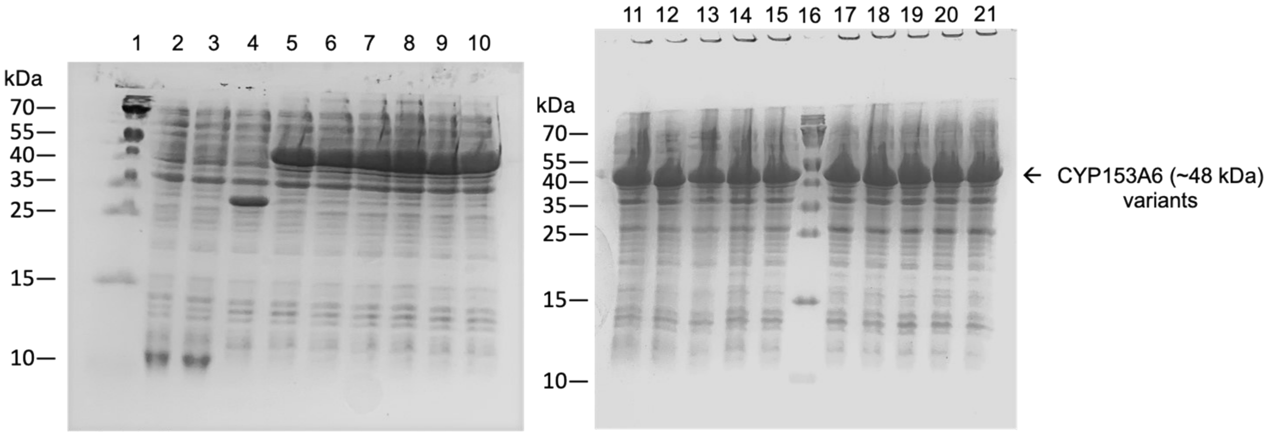

Supplementary Figure S4. SDS-PAGE whole cell lysate analysis of wild type and mutant **2** production strains.

Left:

Lane 1, Protein ladder; Lane 2, pACYC-empty; Lane 3, *Δ*(P450/RED), Lane 4; truncated P450-(*Δ*RED)-Fd (not relevant to this study); Lane 5/6, wild-type CYP153A6; Lane 6, Lane 7, CAO 1.5; Lane 8, CAO 1.2, Lane 9 CAO 1.1; Lane 10, CAO 1.6;

Right:

Lane 11, wild-type CYP153A6; Lane 12, CAO 1.2.1; Lane 13, CAO 1.6:1.4; Lane 14, CAO 1.4; Lane 15, CAO 1.2.2; Lane 16, Protein ladder; Lane17, CAO 1.1:1.2:1.4; Lane 18, CAO 1.2:2.1:1.4; Lane 19, CAO 1.3; Lane 20, CAO 1.6.2.

Note: The stacking gel layer on the left gel is missing. The gel is not cropped.

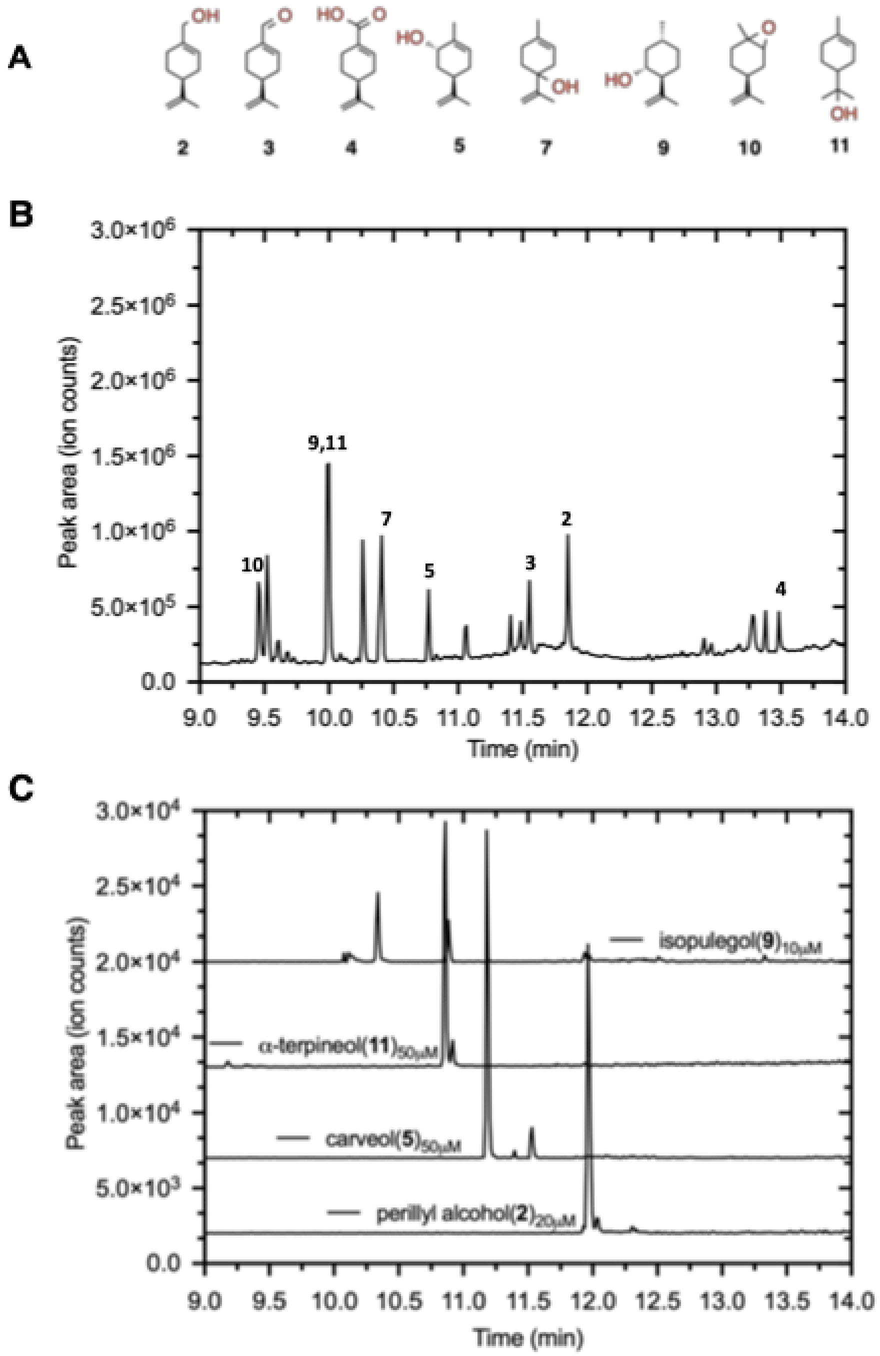

Supplementary Figure S5. GC-MS profiles of hydroxylated limonene derivatives. A) Structures of reference compounds used for the characterization of biosensor effector specificity, including **2**, *(S)*-(-)-perillaldehyde (**3**), *(S)*-(-)-perillic acid (**4**), (-)-carveol (**5**), -(-)-terpineol (**7**), isopulegol (**9**), (-)-limonene oxide (**10**), and (-)-α-terpineol (**11**). **B**) Representative total ion chromatogram (TIC) of the mixture of **2-11**. **C**) Extracted ion chromatograms (EICs) of individual compounds **2**, **5**, **9**, and **11** were used to aid peak assignment in **B**.

Supplementary Methods

Construction of pSENSE-CymR biosensor circuit plasmid

The plasmid pSENSE-CymR biosensor was constructed by cloning the operator sequence cuO into pSENSE2 using primers 1-2 (**Supplementary Table S2**) and the *Avr*II and *Spe*I restriction sites to generate pSENSE3. The CymR fragment, which was codon-optimized for expression in *E. coli*, was amplified using primers 3-4 (**Supplementary Table S2**). The amplified products were analyzed on a 0.8% agarose gel, extracted using a Monarch Gel Extraction Kit (New England Biolabs), digested with *Avr*II and *Spe*I, and ligated for 18 h at 16 °C. The mixture was then transformed into chemically competent *E. coli* DH5α cells. The transformation mixture was plated on LB agar containing ampicillin (100 µg/mL) and incubated for 16 h at 37 °C. A single colony was cultured in 3 mL of ampicillin-treated LB for 16 h at 37 °C with shaking at 250 rpm, then plasmid DNA was isolated using the Monarch Spin Plasmid Miniprep Kit according to the instructions.

Structure-guided saturation mutagenesis of CymR.

Saturation libraries of CymR were constructed using the QuikChange II Site-Directed Mutagenesis protocol with the pSENSE-Cym template and the corresponding primers (see entries 5-14 in **Supplemental Table S1**. The libraries were then transformed into *E. coli* TOP10 and individual colonies from each library (~200) were selected for screening. Individual colonies were picked and used to inoculate a single well of a 96-well plate containing 500 µL of LB media supplemented with Amp (100 µg/mL). The cultures were grown for 5 h at 37 °C with shaking at 350 rpm, at which point, 10 µL of the culture was transferred into a well containing fresh 485 µL of LB media supplemented with Amp (100 µg/mL) and 5 µL of the desired isoprenoid (2.5 mM final concentration) or DMSO. The assay was run for ~16 h at 37 °C with shaking at 350 rpm. The cultures were then centrifuged at 4°C, 3,000 rpm for 10 min, and the supernatant was discarded. The cell pellet was then resuspended in 1 mL of phosphate-buffered saline (PBS). Then, 100 µL of the cell suspension was transferred to clear flat-bottom and black flat-bottom 96-well plates for analysis of the optical density (OD_{600}) and fluorescence (ex 485 nm/ em 510 nm). The fluorescence intensity was divided by the OD_{600} to yield a normalized sfGFP fluorescence value (RFU). Mutants were evaluated by comparing the normalized fluorescence of the wells containing the desired terpene to those containing DMSO (ON/OFF) to get relative fold-activation.

Construction of the prototype 2-producing *E. coli* strain.

The CymR gene fragment (**Supplemental Table S2**) was purchased from Twist Bioscience (San Francisco, CA). The CYP153 insert and linearized pACYCDuet-RED-Fd backbone were amplified by PCR from parent plasmid pACYCDuet-P450-RED-Fd using primers 15/16 and 17/18, respectively (**Supplementary Table S1**). Reactions were performed using Q5 High-Fidelity DNA Polymerase (New England Biolabs) according to the manufacturer's protocol. PCR conditions were as follows: initial denaturation at 98 °C for 30 s; 25 cycles of 98 °C for 10 s, 52 °C (insert) or 52 °C (backbone) for 15 s, and 72 °C for 40 s (insert) or 2.4 min (backbone); followed by a final extension at 72 °C for 10 min and hold at 4 °C. PCR products were treated with *Dpn*I at 37 °C for 1 h, followed by heat inactivation at 80 °C for 20 min. Products were analyzed by electrophoresis on a 0.8% agarose gel and purified using the Monarch Gel Extraction Kit (New England Biolabs). Purified PCR products were quantified and stored at -20 °C.

Error-prone PCR of wild-type CYP153A6.

Random mutagenesis of the P450 gene was performed using error-prone PCR to generate a variant library using the PCR insert (~1.3 kb, 30 ng/µl) product mentioned above. The reaction was carried out with Mutazyme II DNA polymerase (New England Biolabs) under mutagenic conditions: 3 µl Mutazyme II buffer, 0.7 µl dNTPs (100 mM), and 1.0 µl Mutazyme II DNA polymerase. The 25 µL PCR reaction contained 1 ng of plasmid DNA as template and 0.5 µM of the primer pairs 15/16 and 17/18. PCR conditions were: 95 °C for 2 min; 34 cycles of 95 °C for 30 s, 52 °C for 30 s, 72 °C for 40 s; followed by 72 °C for 10 min. The PCR product was analyzed by electrophoresis on a 0.8% agarose gel and purified using the Monarch Gel Extraction Kit (New England Biolabs).

Purified error-prone and linearized pACYCDuet-RED-Fd PCR products were assembled using NEBuilder HiFi DNA Assembly Master Mix (New England Biolabs) at 50 °C for 1 h at a 4:1 molar ratio of insert to backbone in a 10 µL reaction. The Gibson-assembled library (8 µL) was transformed into 50 µL chemically competent *E. coli* TOP10 and recovered in 950 µL SOC medium at 37 °C for 2 h. After recovery, a 50 µL transformation volume was split into two: 25 µL was plated on LB agar containing kanamycin (50 µg/mL), and the remaining 25 µL was used to inoculate 3 mL LB (kanamycin, 50 µg/mL) for overnight growth at 37 °C with shaking at 250 rpm for 16 h. Ten colonies from the agar plate were picked for plasmid extraction and Sanger

sequencing to evaluate the mutation rate and sequence diversity of the library. The overnight culture was used for plasmid miniprep and carried forward to generate expression-ready competent cells. The DNA plasmid of the wild-type and S77L CymR biosensor was transformed into separate 50 µL of *E. coli* BL21(DE3) competent cells that contained wild-type and library P450 system variants. The transformation process involved a heat shock step at 42 °C for 50 s, followed by a recovery phase in 950 µL of SOC medium at 37 °C for 2 h while shaking at 250 rpm. After recovery, a 25 µL aliquot of the transformation mixture was plated on LB agar supplemented with a 1:1 ratio of ampicillin and kanamycin at 50 µg/mL. The plates were then incubated for 16 h at 37 °C.

GC-MS Analysis of wild-type and variant CYP153A6 strains.

A 300 µL aliquot of culture was transferred into separate Eppendorf tubes, and an equal volume of ethyl acetate was added. This was followed by centrifugation at 4,816 x *g* for 10 min at 25 °C. The organic layer was removed, dried over $MgSO_4$, and transferred to glass tubes for GC-MS analysis. GC-MS analysis was conducted on an Agilent 8860 using a J&W CycloSil-B, with a 30 m x 250 µm x 0.25 µm capillary column. Helium (99.99% purity) was used as a carrier gas at a flow rate of 1.2 mL/min. The oven was programmed to start at 50 °C for 3 min, then increase at 25 °C/min to 100 °C, followed by a 10 °C/min increase to 140 °C, and finally a 20 °C/min increase to 250 °C. The injection port was set at 250 °C on pulsed splitless mode. The injection volume was 1 µL. The EI mode 70 eV. The mass data were acquired after a 4.2 min solvent delay in scan mode, ranging from 30 to 500 *m*/*z*.

Site-directed mutagenesis of CYP153A6.

Site-directed mutagenesis was used to create single, double, and triple CYP153A6 variants, resulting in a total of nine rationally designed mutants: A83T (CAO 1.2.1), L282I (CAO 1.2.2), R280L (CAO 1.6.1), H2343L (CAO 1.3.1), A282T (CAO 1.6), L282I/T346I (CAO 1.2.2:1.4), R280L/T346I (CAO 1.6:1.4), L282I/D337N/I348N (CAO 1.2.2:1.7.2), and A83T/D92H/T346I (CAO 1.1:1.2:1.4). Primers designed to introduce single or combinatorial mutations were designed using the NEBaseChanger tool (New England Biolabs).

The wild-type P450-system plasmid was used as the template to generate five single-point mutants: CAO 1.2.1, CAO 1.3.1, CAO 1.2.2, CAO 1.6.1, and CAO 1.6.2 using primer pairs 19/20, 23/24, 25/26, 27/28, and 29/30, respectively (Supplementary Table S1). PCR reactions were performed in a volume of 25 µL containing Q5 Hot Start High Fidelity 2X master mix (12.5 µL), DNase-free water (9 µL), forward and reverse primer mixture (2.5 µL, 10 µM), and 2 ng of

template. Thermocycler conditions were as follows: 98 °C, 30 s; [98 °C, 10 s; 61 °C, 15 s; 72 °C, 3.2 min] cycle step 2-4 25X, 72 °C, 10 min, and hold at 4 °C.

Mutant CAO 1.4 was used as a template to generate three variants, CAO 1.2.2:1.4, CAO 1.6:1.4, and A83T/T346I (CAO 1.2:1.4), using primer pairs 7/8, 25/26, and 27/28, respectively (**Supplementary Table S1**). The triple mutant CAO 1.1:1.2:1.4 was constructed by introducing the A83T mutation into the CAO 1.2:1.4 using primer pairs 19/20. PCR conditions were identical to those described above.

To construct CAO 1.2.2:1.7.2, mutant CAO 1.2.2 was used as a template. Primer pair 31/32 introduced D337N substitution to create an intermediate variant L282I/D337N, which was then used as a template to introduce the I348N mutation. The PCR parameters were as follows: 98 °C, 30 s; [98 °C, 10 s; 63 (D337N), 56 (I348N) °C, 15 s; 72 °C, 3.2 min] cycle step 2-4 25X, 72 °C, 10 min, and hold at 4 °C. The PCR product was digested with *Dpn*I at 37 °C for 1h, then heated to 80 °C for 20 min, analyzed on a 0.8% agarose gel, and extracted using a Monarch gel extraction kit.

All PCR products were digested with *Dpn*I at 37 °C for 1 h, then heated to 80 °C for 20 min, analyzed on a 0.8 % agarose gel, and extracted using a Monarch gel extraction kit. The ligated reaction (4 µL) was transformed into 50 µL chemically competent *E. coli* TOP10 and recovered in 950 µL SOC medium at 37 °C for 1 h. The 25 µL of resuspended transformation mixture was plated on LB agar containing kanamycin (50 µg/mL) and incubated for 16 h at 37 °C.

Two colonies per construct were cultured in 3 mL LB medium containing kanamycin (50 µg/mL) at 37 °C with shaking at 250 rpm for 16 h. Plasmids were isolated using the Monarch spin plasmid miniprep kit, and all mutations were verified by Sanger sequencing. Verified constructs were subsequently transformed into *E. coli* BL21 Star (DE3) and used for protein expression and downstream productivity analysis via GC-MS parameters described above.
